# Oral insulin immunotherapy in children at risk for type 1 diabetes in a randomised controlled trial

**DOI:** 10.1007/s00125-020-05376-1

**Published:** 2021-01-30

**Authors:** Robin Assfalg, Jan Knoop, Kristi L. Hoffman, Markus Pfirrmann, Jose Maria Zapardiel-Gonzalo, Anna Hofelich, Anne Eugster, Marc Weigelt, Claudia Matzke, Julia Reinhardt, Yannick Fuchs, Melanie Bunk, Andreas Weiss, Markus Hippich, Kathrin Halfter, Stefanie M. Hauck, Jörg Hasford, Joseph F. Petrosino, Peter Achenbach, Ezio Bonifacio, Anette-Gabriele Ziegler

**Affiliations:** 1 Institute of Diabetes Research, Helmholtz Zentrum München, German Research Center for Environmental Health, Munich-Neuherberg, Germany; 2 Forschergruppe Diabetes, Technical University Munich, at Klinikum rechts der Isar, Munich, Germany; 3 German Center for Diabetes Research (DZD), Munich, Germany; 4 Alkek Center for Metagenomics and Microbiome Research, Department of Molecular Virology and Microbiology, Baylor College of Medicine, Houston, TX USA; 5 Institute for Medical Information Processing, Biometry, and Epidemiology, Ludwig-Maximilians-University Munich, Munich, Germany; 6 Technische Universität Dresden, Center for Regenerative Therapies Dresden, Dresden, Germany; 7 Paul Langerhans Institute Dresden of the Helmholtz Center Munich at University Hospital Carl Gustav Carus and Faculty of Medicine, TU Dresden, Dresden, Germany; 8 Research Unit Protein Science, Helmholtz Zentrum München, German Research Center for Environmental Health, Munich-Neuherberg, Germany; 9 Institute for Diabetes and Obesity, Helmholtz Diabetes Center at Helmholtz Zentrum München, Munich-Neuherberg, Germany

**Keywords:** Autoimmunity, Insulin, Oral immunotherapy, Primary prevention, Type 1 diabetes

## Abstract

**Aims/hypothesis:**

Oral administration of antigen can induce immunological tolerance. Insulin is a key autoantigen in childhood type 1 diabetes. Here, oral insulin was given as antigen-specific immunotherapy before the onset of autoimmunity in children from age 6 months to assess its safety and immune response actions on immunity and the gut microbiome.

**Methods:**

A phase I/II randomised controlled trial was performed in a single clinical study centre in Germany. Participants were 44 islet autoantibody-negative children aged 6 months to 2.99 years who had a first-degree relative with type 1 diabetes and a susceptible *HLA DR4-DQ8*-containing genotype. Children were randomised 1:1 to daily oral insulin (7.5 mg with dose escalation to 67.5 mg) or placebo for 12 months using a web-based computer system. The primary outcome was immune efficacy pre-specified as induction of antibody or T cell responses to insulin and measured in a central treatment-blinded laboratory.

**Results:**

Randomisation was performed in 44 children. One child in the placebo group was withdrawn after the first study visit and data from 22 insulin-treated and 21 placebo-treated children were analysed. Oral insulin was well tolerated with no changes in metabolic variables. Immune responses to insulin were observed in children who received both insulin (54.5%) and placebo (66.7%), and the trial did not demonstrate an effect on its primary outcome (*p* = 0.54). In exploratory analyses, there was preliminary evidence that the immune response and gut microbiome were modified by the *INS* genotype Among children with the type 1 diabetes-susceptible *INS* genotype (*n* = 22), antibody responses to insulin were more frequent in insulin-treated (72.7%) as compared with placebo-treated children (18.2%; *p* = 0.03). T cell responses to insulin were modified by treatment-independent inflammatory episodes.

**Conclusions/interpretation:**

The study demonstrated that oral insulin immunotherapy in young genetically at-risk children was safe, but was not associated with an immune response as predefined in the trial primary outcome. Exploratory analyses suggested that antibody responses to oral insulin may occur in children with a susceptible *INS* genotype, and that inflammatory episodes may promote the activation of insulin-responsive T cells.

**Trial registration:**

Clinicaltrials.gov NCT02547519

**Funding:**

The main funding source was the German Center for Diabetes Research (DZD e.V.)

**Graphical abstract:**

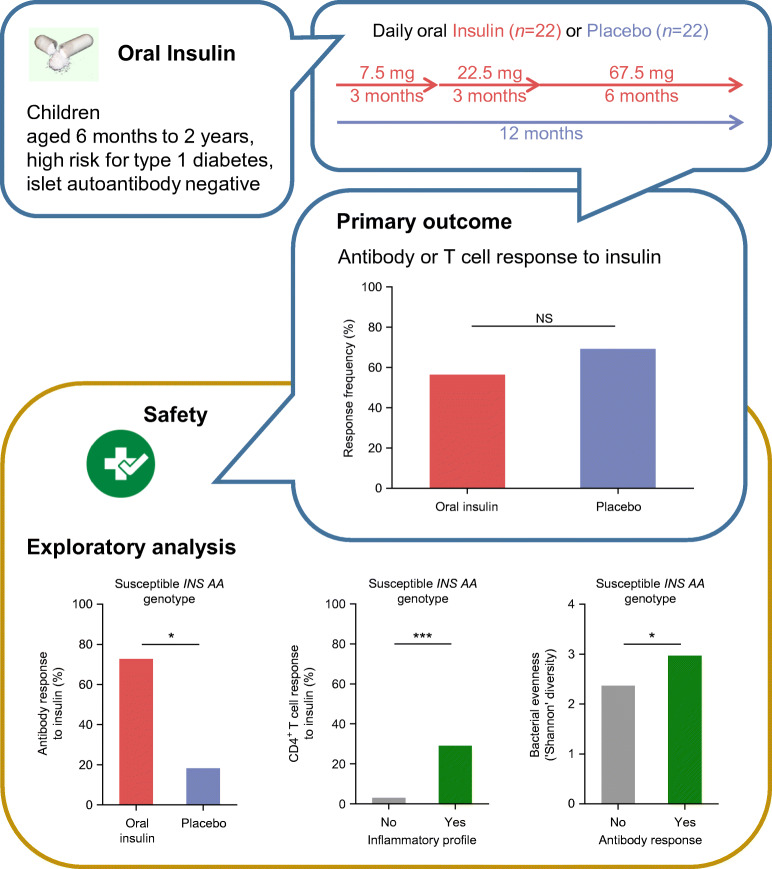

**Supplementary Information:**

The online version contains peer reviewed but unedited supplementary material available at 10.1007/s00125-020-05376-1.

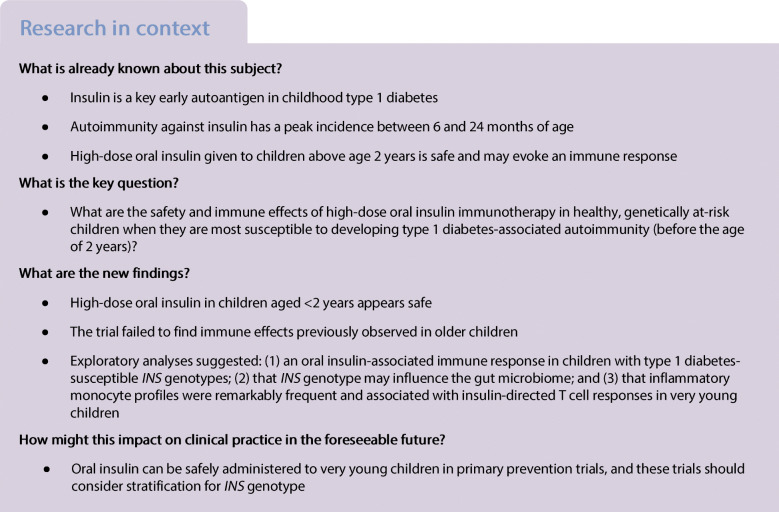



## Introduction

Type 1 diabetes results from an autoimmune destruction of insulin-producing beta cells in the pancreatic islets of Langerhans, and is characterised by circulating islet autoantibodies to beta cell antigens [1, 2]. Insulin is a key early autoantigen in childhood diabetes [3, 4]. Autoimmunity against insulin often appears in genetically susceptible children aged 9 months to 3 years, with a peak incidence at 9–12 months of age [5–7], and this loss of immune tolerance to insulin often leads to type 1 diabetes [8, 9]. Immune tolerance to insulin is influenced by the HLA DRB1*04-DQB1*0302 haplotype [8] and allelic variations in *INS*, the gene that encodes insulin [10–12], via mechanisms involving thymic T cell deletion [13, 14].

Controlled exposure to antigen leads to protection against immune-mediated diseases such as childhood allergy [15] and in animal models of autoimmunity [16]. In type 1 diabetes, attempts have been made to reduce disease risk in individuals with established autoimmunity by administration of autoantigen orally [17, 18], intranasally [19, 20], intravenously or subcutaneously [21, 22]. Treatment-associated immune effects such as increases in antibody titres [20–22] and changes in CD4^+^ T cell responses to administered autoantigen were observed in some of these studies [20], indicating that administration could lead to immune modulation. Although none of these trials achieved their primary outcomes of diabetes prevention, beneficial treatment effects were observed in exploratory analyses of subgroups within the oral insulin immunotherapy trials [17, 18].

We reasoned that, similar to peanut allergy [15], the efficacy of antigen-specific immune therapy to prevent autoimmune disease would improve if treatment was started early in life and as a primary prevention therapy before individuals become autoantibody positive [23]. We previously demonstrated that daily oral administration of high doses (67.5 mg) of insulin to children with a genetic risk of type 1 diabetes did not induce unwanted hypoglycaemia and was associated with the induction of low-affinity antibodies against insulin and insulin-responsive CD4^+^ T cells with features of regulation [24]. These treatment-associated immune responses were not typical of autoimmune diabetes [8, 25]. We, therefore, inferred that the treatment was likely to be safe and capable of inducing an immune response that might protect against the development of type 1 diabetes. While these earlier findings are an important proof of concept, they were obtained in a small number of children aged 2–7 years, which is after the period of greatest susceptibility to insulin autoimmunity and, therefore, late for primary prevention of islet autoimmunity. Here, we report the Pre-POInT-early RCT in children aged 6 months to 2 years, which represents the first intervention with autoantigen at this very early age and, therefore, uniquely analyses overall safety, immune responses and effects of exposure to exogenous autoantigen during peak susceptibility. Children in this age group undergo a transition from maternally derived immunity to acquired protection through exposure to vaccinations and infectious agents [26], and large changes in the immune repertoire and the gut microbiome. Daily exposure of the mucosal immune system to a key autoantigen in genetically susceptible children during this period presents a rare opportunity to assess the interplay between these factors in eliciting immune responses.

The Pre-POInT-early trial had four objectives. First, to determine the safety of daily oral insulin administration in very young children with high genetic susceptibility for type 1 diabetes; second, to determine whether the previously observed antibody and CD4^+^ T cell responses to oral insulin could be observed in younger children; third, to explore interactions between oral insulin therapy and *INS* genotype and microbiome; and, fourth, to investigate immune changes and events that may influence autoimmunity during this period of high susceptibility.

## Methods

### Participants

The Pre-POInT-early study was a randomised, placebo-controlled, double-blind, single-centre, pilot phase II clinical study (Clinicaltrials.gov registration no. NCT02547519). Participants were recruited between December 2015 and December 2016. Follow-up visits were completed in December 2017. Children were eligible if they were aged 6 months to 2.99 years; seronegative for autoantibodies to insulin (IAA), GAD (GADA), insulinoma-associated antigen 2 (IA-2A) and zinc transporter-8 (ZnT8A); and at high genetic risk of developing type 1 diabetes. High genetic risk was defined by a first-degree relative with type 1 diabetes diagnosed before age 40 years and an HLA genotype that included the HLA DRB1*04-DQB1*0302 or HLA DRB1*04-DQB1*0304 haplotypes (*DR4-DQ8*), and did not include one of the following alleles or haplotypes: DRB1*11, DRB1*12, DQB1*0602, DRB1*07-DQB1*0303, DRB1*14-DQB1*0503. The study was approved by the Ethikkommission der Fakultät für Medizin der Technischen Universität München (206/15). The parents or legal guardians of each child provided written, informed consent before inclusion in the study. The study was performed in compliance with the current version of the Helsinki declaration.

### Intervention and procedures

**Fig. 1:**
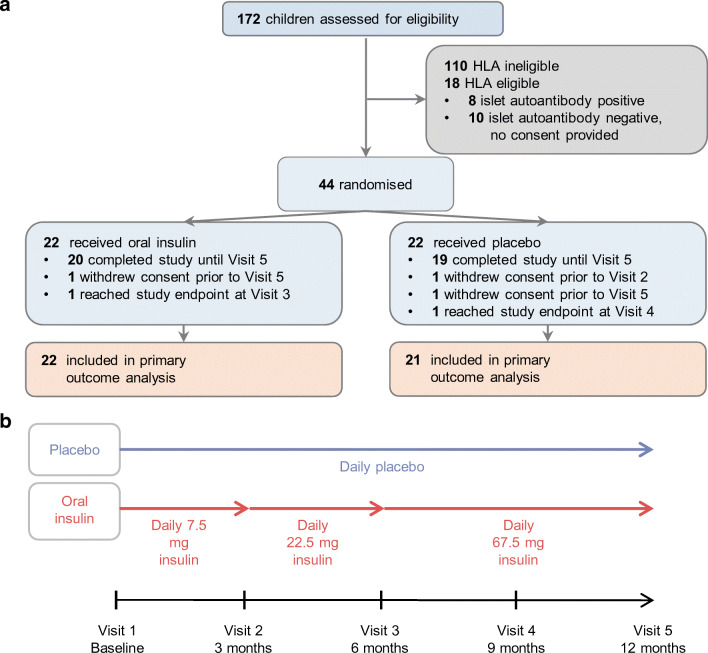
Schematics of participant disposition, design and treatment groups. (**a**) Disposition of the participants. All children had a first-degree family history of type 1 diabetes. Study endpoint was the development of persistent antibodies to GAD, IA-2 or ZnT8. (**b**) Study design and treatment groups

Children were randomised 1:1 to receive oral insulin or placebo daily for a period of 12 months, and parents were instructed how to administer the study drug. Investigators and participants were masked to treatment allocation. See the electronic supplementary material (ESM) Methods for further details. Children in the oral insulin group received 7.5 mg of insulin for 3 months, then 22.5 mg for 3 months, and finally 67.5 mg for 6 months (Fig. 1b). Follow-up visits were scheduled at 3, 6, 9 and 12 months after starting treatment. Blood samples and saliva were collected at each visit to determine islet autoantibodies, immune responses to insulin, lymphocyte and monocyte subsets, and salivary IgA antibodies to insulin using radio binding assays [24, 27–31], dye dilution cell proliferation assays (ESM Fig. 1) [24] and FACS analyses, respectively (see ESM Methods). Single-cell gene expression profiles were obtained for CD4^+^ T cells responding to insulin (see ESM Methods). At the baseline, 3 month and 6 month visits, blood samples were collected before (−10 min) and 30, 60, 90 and 120 min after study drug intake to measure blood glucose, insulin and C-peptide (see ESM Methods). Blood cell counts, blood chemistry, electrolytes, IgE and plasma markers of inflammation were measured at baseline and 12 months (see ESM Methods). Stool samples were collected to assess the microbiome at baseline, 6 months and 12 months (see ESM Methods). The *INS* genotype of the children was determined (see ESM Methods). Medication adherence was assessed by family self-reporting of daily capsule administration using adherence sheets. Adverse events were recorded throughout the study (see ESM Methods). Children reached study endpoint and stopped treatment if they developed persistent islet autoantibodies (GADA, IA-2A or ZnT8A) or clinical diabetes. All data except the plasma markers of inflammation were submitted before the data hard lock which was signed on 18 July 2018. Data were unblinded by the independent study statistician on 24 July 2018.

### Study outcome

The primary immune efficacy outcome was an immune response to insulin, defined as an increase in any one or more of the following: serum IgG antibodies to insulin, salivary IgA antibodies to insulin, serum IAA or a CD4^+^ T cell response to insulin. Additional secondary and exploratory outcomes included the gene expression profile of CD4^+^ T cells responding to insulin, the antibody and CD4^+^ T cell responses in children with the *INS* AA genotype, and the stool microbiome. Post hoc analyses included T lymphocyte and monocyte subset frequencies, and plasma inflammatory markers.

### Statistics

#### Sample size

The null hypothesis was that the probability of developing an antibody and/or T cell response to insulin in the oral insulin group equals the probability of developing an antibody and/or T cell response in the placebo group. Based on results of the Pre-Primary Oral Insulin Trial (Pre-POInT) study [24], response rates of 20% in the placebo group and 67% in the oral insulin group were assumed. Accordingly, enrolment and randomisation of 44 children 1:1 to two treatment groups was expected to be sufficient to reject the null hypothesis with a two-sided Fisher’s exact test at a significance level of 0.05 and a power of 80% and 10% dropout. The sample size estimation was performed using PS Power and Sample Size Calculations software version 3.0.43 [32].

#### Statistical comparisons

To compare continuous variables between the two independent groups, we used the Mann–Whitney *U* test with normal approximation. The Kruskal–Wallis test was applied when the number of groups was larger than two. Differences in categorical variables between the groups were assessed using Fisher’s exact test. Unless otherwise indicated, continuous variables are reported as the median and IQR. Time-to-event variables were summarised using Kaplan–Meier plots and compared with the logrank test. Additional analyses compared the immunological outcomes in children with the *INS* AA genotype and treatment effects on the stool microbiome (see ESM Methods). The significance level of two-sided *p* values was 0.05 for all statistical tests. Point estimates are given together with the 95% CIs. The analysis for this paper was generated using SAS software 9.4 (SAS Institute, Cary, NC, USA), R software (2020); https://www.R-project.org/), the R software packages ‘vegan’ and ‘ggplot2’ [33, 34] and GraphPad Prism software 7.05 (GraphPad Software, San Diego, California USA).

## Results

### Participant enrolment and treatment

In total, 172 infants aged 6 months to 2 years with a first-degree family history of type 1 diabetes were screened (Fig. 1a). Of these, 54 were eligible based on their *HLA DRB1-DQA1-DQB1* genotype and the lack of islet autoantibodies. Consent to participate was provided for 44 children (17 girls), who were randomised at a median age of 1.1 years (IQR, 0.8–1.7 years). Randomised groups were reasonably balanced with respect to baseline characteristics (Table 1). All of the randomised children received at least one dose of their allocated treatment. The cumulative insulin exposure was 66.7, 67.2 and 126.2 months for the 7.5 mg, 22.5 mg and 67.5 mg doses, respectively (ESM Table 1). One child in the placebo group was withdrawn before attending the 3 month follow-up visit; no adverse event reporting or immune data were available from this child. Median adherence to the allocated treatments was 98.0% for placebo and 96.9% for oral insulin (ESM Table 1). Of 220 planned study visits, 212 (96.4%) were completed.

**Table 1:** Baseline characteristics of children enrolled in the Pre-POInT-early study

Characteristic	Placebo	Oral insulin
Participants, n	22	22
Girls, n (%)	10 (45.5)	7 (31.8)
Age, median (IQR); years	1.2 (0.9–1.9)	1.0 (0.8–1.4)
Weight, median (IQR); kg	10.5 (9.3–12.8)	9.6 (8.3–11.7)
Height, median (IQR); cm	78.0 (73.0–87.0)	74.0 (70.0–82.0)
First-degree T1D relative (n)		
Mother	8	7
Father	6	6
Sibling	4	5
Multiplex^a^	4	4
*HLA* genotype (n)		
DRB1*03/DR4-DQ8	6	5
*DR4-DQ8/DR4-DQ8*	3	3
*DR4-DQ8/x*^b^	13	14
*INS* VNTR genotype (n)		
*A/A*	11	11
*A/T*	8	11
*T/T*	1	0

^a^Multiplex indicates that a child has at least two first-degree relatives with type 1 diabetes

^b^x = non-DRB1*03, non-DRB1*04

T1D, type 1 diabetes; VNTR, variable number tandem repeat

### Participant safety

**Fig. 2:**
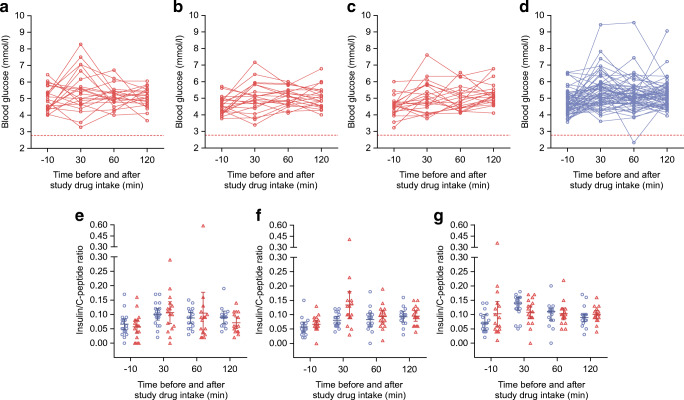
Blood glucose concentration and insulin/C-peptide ratio over time. (**a**–**d**) Blood glucose concentrations were measured in children before and after intake of oral insulin at a dose of 7.5 mg at baseline visit 1 (**a**; *n* = 22), 22.5 mg at 3 months visit 2 (**b**; *n* = 22), and 67.5 mg at 6 months visit 3 (**c**; *n* =22), or placebo at visits 1–3 (**d**; *n* = 63). The concentrations for individual children are connected by lines. The dashed line indicates the threshold for hypoglycaemia at 2.78 mmol/l (**a**–**d**). (**e**–**g**) The insulin/C-peptide ratio is plotted for each time point at visit 1 (**e**), visit 2 (**f**) and visit 3 (**g**) for children receiving oral insulin (red triangles) or placebo (blue circles)

Oral insulin therapy was well tolerated with no evidence of treatment-related hypoglycaemia. All blood glucose concentrations measured within 2 h after the first dose of placebo or oral insulin, or the first dose after each dose escalation, were >2.78 mmol/l, except for one instance in a child in the placebo group (Fig. 2a–d). Blood glucose, insulin or C-peptide values (ESM Table 2); the insulin/C-peptide ratio (Fig. 2e–g); and the areas under the concentration–time curves for glucose, insulin or C-peptide (ESM Table 2) were similar between the placebo and insulin groups. Persistent GADA, IA-2A or ZnT8A (study endpoint) developed 6.9 months after randomisation in one child in the oral insulin group and after 9.5 months in one child in the placebo group. Blood counts and blood chemistry were similar between the two groups (ESM Table 3). A total of 114 adverse events were reported over a cumulative exposure period of 21.1 years in 21/21 children in the placebo group (5.64 events per year), and 181 adverse events were reported over a cumulative exposure period of 21.7 years in 22/22 children in the oral insulin group (8.38 events per year) (ESM Table 4). There were six serious adverse events, four in the oral insulin group and two in the placebo group, none of which were considered related to the study drug. By system organ class, the frequency of skin and subcutaneous tissue disorders was greater in the oral insulin group (12 events in eight children) than in the placebo group (one event in one child; *p* = 0.01; ESM Fig. 2). See the ESM Results for further details on laboratory findings, adverse events and protocol adherence.

### Immunological response to daily oral insulin administration

**Fig. 3:**
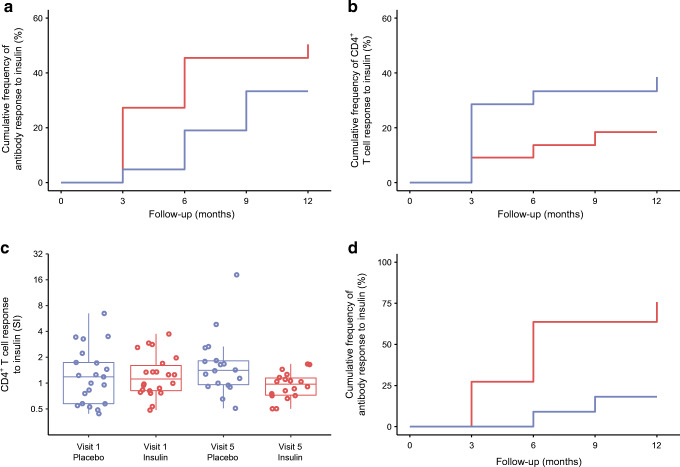
Responses to treatment and analysis of responses. (**a**, **b**) Immune response to oral insulin or placebo. Kaplan–Meier analysis of a positive antibody response to insulin (**a**) and CD4^+^ T cell response to insulin (**b**) as defined by the primary outcome criteria in children who received placebo (blue line; *n* = 21) or oral insulin (red line; *n* = 22). The follow-up time is calculated from the first day of treatment (**a**, **b**). (**c**) CD4^+^ T cell response to insulin calculated as the SI relative to medium control at baseline (visit 1) and at 12 months (visit 5) in children who received placebo (blue circles; *n* = 21 at baseline, *n* = 18 at 12 months) or oral insulin (red circles; *n* = 22 at baseline, *n* = 18 at 12 months). (**d**) Kaplan–Meier analysis of a positive antibody response to insulin as defined by the primary outcome criteria in children with the *INS* AA genotype who received placebo (blue line; *n* = 11) or oral insulin (red line; *n* = 11; *p* = 0.0085)

The primary outcome was based on findings from the previous Pre-POInT trial [24] as a positive antibody or CD4^+^ T cell response to insulin. The primary outcome was observed in 26/43 (60.5%) children at 3 (*n* = 13), 6 (*n* = 9) or 9 months (*n* = 4) after randomisation (Table 2), including 14/21 (66.7%) in the placebo group and 12/22 (54.5%) in the oral insulin group (*p* = 0.54). The cumulative frequencies of antibody responses to insulin were 33.4% in the placebo group and 50.4% in the oral insulin group (*p* = 0.18; Fig. 3a). Responses included an increased IgG binding to insulin (seven in placebo group and nine in oral insulin group) and a salivary IgA response to insulin (one in placebo group and two in oral insulin group). The cumulative frequencies of the positive CD4^+^ T cell responses to insulin were 38.5% in the placebo group and 18.4% in the oral insulin group (*p* = 0.15; Fig. 3b). CD4^+^ T cell responses to insulin at 12 months were lower in the oral insulin group (median stimulation index [SI], 0.97; IQR, 0.71–1.21) than in the placebo group (median SI, 1.41; IQR, 0.94–2.2; *p* = 0.014; Fig. 3c).

**Table 2:** Primary outcome response to insulin

Treatment group	Positive antibody outcome (*n*)	Positive CD4^+^ T cell outcome (*n*)	Positive trial outcome (*n*)
All children (*N* = 43)			
Placebo (*n* = 21)	7 (33.3)	8 (38.1)	14 (66.7)
Oral insulin (*n* = 22)	11 (50.0)	4 (18.2)	12 (54.5)
*INS* AA genotype (*n* = 22)			
Placebo (*n* = 11)	2 (18.2)	5 (45.5)	7 (63.6)
Oral insulin (*n* = 11)	8 (72.7)	4 (36.4)	9 (81.8)

Data are given as *n* (%)

In conclusion, the study failed to demonstrate an effect on its predefined primary outcome. As compared with the previous Pre-POInT study in older children, the responses to insulin were more frequently observed in the placebo group of this study (66.7% vs 20% [24]; *p* = 0.02), but not in the oral insulin groups (54.5% vs 60%). This suggests frequent activation of immune responses to insulin in very young genetically susceptible children in the study.

### The *INS* genotype was associated with immunological responses to treatment

Autoimmunity against insulin is more frequent in children with the susceptible *INS* AA genotype [12, 35]. Therefore, an exploratory analysis of the immune response to insulin stratified for *INS* genotype was included in the study analysis plan. An antibody response to insulin was observed in 10/22 children with the susceptible *INS* AA genotype, including 2/11 (18.2%) children in the placebo group and 8/11 (72.7%) children in the oral insulin group (*p* = 0.03). Cumulative frequencies of antibody responses at 12 months were 18.2% (95% CI 0.1%, 40.4%) in placebo and 75.8% (95% CI 48.8%, 99.9%) in oral insulin groups (*p* = 0.0085; Fig. 3d, ESM Table 5). The majority (*n* = 7) of children with the susceptible *INS* AA genotype in the oral insulin group showed an antibody response by 6 months of treatment. An interaction between *INS* genotype and treatment was statistically tested using the Cox proportional hazards model and was observed for an antibody response (*p* = 0.024). Age was inversely associated with the antibody response in this model (*p* = 0.032). Unlike the antibody responses, the frequency of T cell responses to insulin in children with the *INS* AA genotype was not different between the placebo group (5/11) and the oral insulin group (4/11; *p* > 0.99; ESM Table 5). These results suggest that oral insulin may induce an antibody response in very young children with a susceptible *INS* genotype in this study.

### Age, *INS* genotype and treatment are associated with microbiome alterations

**Fig. 4:**
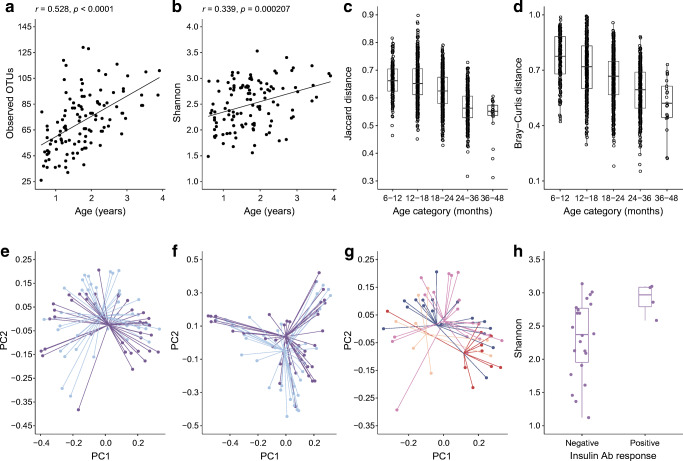
Microbiome alterations in relation to age, *INS* genotype and treatment. (**a**, **b**) Alpha diversity in relation to age over the time of study participation in samples from baseline (*n* = 40), 6 months (*n* = 40) and 12 months (*n* = 35); shown are richness (observed OTU) (**a**) and evenness (Shannon) (**b**). (**c**, **d**) Beta diversity in relation to age over time of study participation (baseline, 6 months, 12 months); shown are Jaccard distance (**c**; *p* < 0.0001) and Bray–Curtis distance (**d**; *p* < 0.0001). Each dot represents the distance between two samples within the age range (**c**, **d**). (**e**–**g**) Beta diversity differences by PCoA (baseline, 6 months, 12 months); shown are Jaccard distance in children with the *INS* AA genotype (purple dots and lines) or the *INS* AT or TT genotype (bright-blue dots and lines) (**e**; *p* = 0.0258), Bray–Curtis distance in children with the *INS* AA genotype (purple dots and lines) or the *INS* AT or TT genotype (bright-blue dots and lines) (**f**; *p* = 0.0422), and Jaccard distance in children with the *INS* AA genotype who received placebo (dark-blue dots and lines) or oral insulin (red dots and lines), and in children with the *INS* AT or TT genotype who received placebo (bright-orange dots and lines) or oral insulin (fuchsia dots and lines) (**g**; *p* = 0.0069). (**h**) Alpha diversity (Shannon) in children with the *INS* AA genotype who had a negative (*n* = 22 samples) or positive (*n* = 4 samples) antibody response to insulin (6 months, 12 months) (*p* = 0.0395). Unless indicated, the plots include both placebo- and oral insulin-treated children. For treatment-related analyses, only post-baseline samples at 6 and 12 months were included. Ab, antibody; PC1, principal component 1; PC2, principal component 2; PCoA, principal coordinates analysis

It is assumed that the potentially beneficial effects of oral exposure to antigen are via tolerogenic presentation in the oral mucosa. We, therefore, included stool collection and planned and performed separate exploratory and post hoc analyses of the microbiome in the study participants. There was a marked relationship between the age of the children and the alpha and beta diversities of the microbiome. Bacterial richness (observed operational taxonomic units [OTUs]) and bacterial evenness (Shannon diversity) increased with age, and bacterial community metrics (unweighted Jaccard distance and weighted Bray–Curtis distance) converged with age (Fig. 4a–d). Similar findings were observed by whole-genome sequencing (ESM Fig. 3a–d). There were significant changes in the abundances of several phyla and genera over time (ESM Fig. 4a,b).

The alpha and beta diversities of the microbiome were similar between the oral insulin and placebo groups. However, there were bacterial community differences between children with the *INS* AA genotype and children with the AT or TT genotypes, and between the treatment groups after stratification by *INS* genotype. The unweighted and weighted bacterial community metrics differed between children with the *INS* AA genotype and children with the AT or TT genotypes (unweighted Jaccard distance *p* = 0.025; weighted Bray–Curtis distance *p* = 0.042; Fig. 4e,f). The relative abundance of *Bacteroides dorei* was increased in children with the *INS* AA genotype (6.2%) vs children with the AT or TT genotypes (0.4%; *p* = 0.002; ESM Fig. 5a). The unweighted Jaccard distance in children with the *INS* AA genotype who received oral insulin differed as compared with the placebo group and with children with the AT or TT genotypes (*p* = 0.0069; Fig. 4g). Subtle differences in alpha diversity were also observed (ESM Fig. 5b,c). There was also a potential increase in bacterial evenness (Shannon diversity) observed among children with the *INS* AA genotype who showed a positive antibody response to insulin compared with children who showed no antibody response (*p* = 0.04; Fig. 4h).

### Type 1 interferon profiles were frequent and associated with inflammatory markers and events

**Fig. 5:**
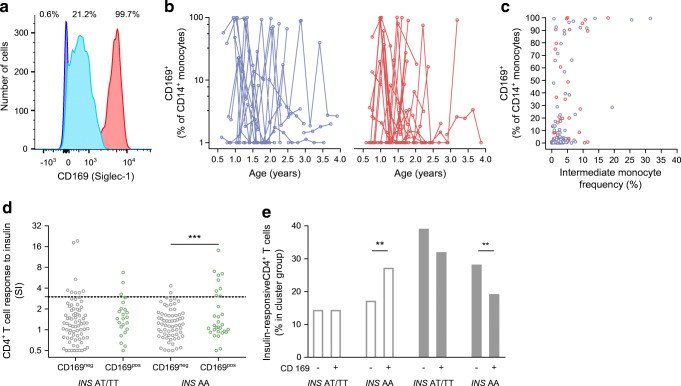
T cell responses to insulin in relation to *INS* genotype and monocyte CD169 expression. (**a**–**d**) Monocyte CD169 expression and CD4^+^ T cell responses to insulin over the time of study participation (baseline, 3, 6, 9, 12 months) in all study participants. (**a**) Representative flow cytometry histograms of CD169 staining intensity on monocytes. Shown are three samples with low (blue; 0.6% positive cells), moderate (light blue, 21.2% positive cells) and high monocyte CD169 expression (red, 99.7% positive cells). A threshold of >5% positive monocytes was used as the threshold for defining monocyte CD169^+^ samples. (**b**) Percentage of CD169^+^ cells out of CD14^+^ monocytes in relation to age over the time of study participation in children who received placebo (blue lines; *n* = 21) or oral insulin (red lines; *n* = 22); plotted on a log scale. (**c**) Correlation between the frequency of CD169^+^ monocytes and the frequency of intermediate monocytes in peripheral blood from children who received placebo (blue circles; *n* = 102 samples) or oral insulin (red circles; *n* = 104 samples; *r* = 0.52, *p* < 0.0001). (**d**) CD4^+^ T cell responses to insulin (SI) in samples from 20 children with the *INS* AT or TT genotype and 22 children with the *INS* AA genotype and stratified by monocyte CD169 expression as negative (CD169^neg^, grey circles; *n* = 148 samples) or positive (CD169^pos^, green circles; *n* = 58 samples) in all study visits; plotted on a log scale. (**e**) The frequencies of insulin-responsive CD4^+^ T cells (*n* = 1036 cells from 22 samples) in the Th1/Th21-like cell clusters 2 and 3 (left; white bars) and the Treg-like clusters 9, 10 and 11 (right; grey bars) according to whether cells were from children with the *INS* AT/TT (*n* = 550 cells) or *INS* AA (*n* = 486 cells) genotype and samples that were monocyte CD169 negative (*n* = 559 cells) or positive (*n* = 477 cells). **p < 0.01, ***p < 0.001

The trial included peripheral blood flow analyses. We found stable cell populations with strong inter-individual differences (Treg, CD8^+^ T cells; ESM Fig. 6a,b) and other cell populations that varied across measurements within children (intermediate monocytes, activated CD8^+^ T cells; ESM Fig. 6c,d). Age was strongly correlated with the frequency of peripheral blood mononuclear cell populations (ESM Fig. 7a,b). We previously found that the two samples exhibiting CD4^+^ T cell responses to insulin among the placebo-treated children in the Pre-POInT study had evidence of a viral inflammatory response. We, therefore, performed post hoc analyses in flow data from the Pre-POInT-early children and frequently observed samples with CD169 (Siglec-1) expression on monocytes (59/208, 28.4%; Fig. 5a,b), an indication of an ongoing type 1 interferon response [36]. This feature was observed in 32/43 (74%) children and was found on multiple samples in 19 (44%) children. Monocyte CD169 expression was associated with the relative frequency of the inflammatory type intermediate (CD14^++^CD16^+^) monocytes (*r* = 0.52; *p* < 0.0001; Fig. 5c), and both of these features were associated with an increased frequency of activated (CD69^+^) T cells (CD169^+^ monocytes, *r* = 0.34, *p* < 0.0001; intermediate monocytes, *r* = 0.65, *p* < 0.0001). In post hoc measurements of inflammatory proteins, monocyte CD169 expression was correlated with the levels of the proinflammatory plasma proteins CXCL10 (*r* = 0.49; *p* = 0.0002), IL-6 (*r* = 0.42; *p* = 0.0038) and IFNg (*r* = 0.39; *p* = 0.0095; ESM Table 6). Monocyte CD169 expression was also associated with decreased gut microbiome richness (*p* = 0.033; ESM Fig. 8a,b) and younger age (*p* = 0.014; ESM Fig. 8c). To determine whether the monocyte CD169 expression corresponded to potential inflammatory episodes in the children, we examined the trial adverse events in the 2 week period before sample collection and observed more adverse events when samples were monocyte CD169^+^ (20/49, 40.8%) than when samples were CD169^−^ (21/118, 17.8%; *p* = 0.0028).

### Type 1 interferon profiles interacted with *INS* genotype to promote CD4^+^ T cell responses to insulin

We speculated that the remarkably frequent inflammatory response may play a role in the pathogenesis of insulin autoimmunity. We found an association between monocyte CD169 expression and positive CD4^+^ T cell responses to insulin (SI > 3; 12/50 CD169^+^ vs 10/146 CD169^−^; *p* = 0.0026), suggesting that the in vitro assay may be affected by the inflammatory state of the child. The association between monocyte CD169 expression and T cell responses to insulin was most evident in children with the susceptible *INS* genotype (Fig. 5d). Among children with the *INS* AA genotype, CD4^+^ T cell responses to insulin were observed in 9/31 (29%) CD169^+^ samples vs 2/68 (3%) CD169^−^ samples (*p* = 0.0004). No difference was observed between the *INS* AA CD169^+^ and *INS* AT/TT CD169^+^ sample groups (*p* = 0.32). A similar relationship was observed for CD8^+^ T cells (ESM Fig. 9). Monocyte CD169 expression was not associated with antibody responses to insulin.

We also examined single-cell transcription profiles of the insulin-responsive CD4^+^ T cells in relation to monocyte CD169 expression. The expression of 76 genes (ESM Table 7) was analysed in 1036 insulin-responsive CD4^+^ T cells from samples with a >3 SI response to insulin. The profiles were distributed in 11 cell clusters (ESM Fig. 10a), including two clusters with features of Th1/Th21-like T cells (*IFNG*, *IL-21*; clusters 2 and 3; *n* = 184 cells), and three clusters with features of Tregs (*FOXP3*, low *CD127* and low cytokine expression; clusters 9, 10 and 11; *n* = 313 cells; ESM Fig. 10b). The distribution across clusters differed between cells from CD169^+^ and CD169^−^ samples (*p* = 1.5 × 10^−10^), between cells from children with the *INS* AA genotype and the AT or TT genotypes (*p* = 4.3 × 10^−7^), and between the CD169^+^ and CD169^−^ samples from children with the *INS* AA genotype (*p* = 0.0003). Among children with the *INS* AA genotype, the CD169^+^ samples contained a higher proportion of insulin-responsive cells in the Th1/Th21-like clusters (88/323; 27.2%) than the CD169^−^ samples (28/163, 17.2%; *p* = 0.0094) and a lower proportion of insulin-responsive cells in the Treg-like clusters (63/323, 19.5% vs 46/163, 28.2%; *p* = 0.0028; Fig. 5e). Altogether, these findings suggest that in vitro presentation of insulin to T cells by antigen-presenting cells will more likely result in a productive Th1/Th21-like T cell response if the cells are from a child with a susceptible *INS* genotype and in an active inflammatory state.

## Discussion

The Pre-POInT-early study is the first to expose very young genetically at-risk children to exogenous autoantigen at an age of peak susceptibility to autoimmunity. It demonstrated that daily oral administration of up to 67.5 mg of insulin to healthy, genetically at-risk, islet autoantibody-negative children at 6 months to 2 years of age was well tolerated without signs of hypoglycaemia. The study did not demonstrate an effect on its primary outcome of immune efficacy defined by the findings in older children [24] as either antibody or T cell responses to insulin. In secondary and exploratory analyses, treatment effects were, however, found for CD4^+^ T cell responses to insulin and in subgroup analyses of children with the susceptible *INS* genotype. Post hoc analyses also revealed remarkably frequent treatment-independent inflammatory episodes with features of type 1 interferon responses in the participants. These inflammatory episodes influenced insulin-directed T cell responses, again in an *INS* gene-associated manner, providing a potential mechanism for the high incidence of islet autoimmunity in early childhood.

Although hypoglycaemia was not previously reported during treatment with oral insulin [17, 18, 24], children <2 years of age have not been exposed to oral insulin. Therefore, the absence of hypoglycaemia at any of the tested doses with a cumulative exposure of >21 years is an important safety outcome. We also found no differences in glucose, insulin and C-peptide over a 2 h period after administration of insulin compared with administration of a placebo. To our knowledge, this is the first study to include comprehensive metabolic measures upon administration of oral insulin in all participating children. These data indicate that oral insulin is unlikely to enter the blood stream, a conclusion that was important for initiating the Primary Oral Insulin Trial (POInT) phase 2b trial in 4–6-month-old infants [37]. Of note, the induction of tolerance by oral antigen is thought to be via antigen uptake in the oral and/or gut mucosa and does not require entry into the blood stream.

As in the Diabetes Prevention Trial–Type 1 (DPT-1) [17], TrialNet [18] and Pre-POInT [24] trials, we observed no signs of allergy or intolerance to orally administered insulin. The frequency of adverse events was not increased in the oral insulin group, except for skin and subcutaneous tissue disorders. This was not observed in larger secondary prevention DPT-1 [17] and TrialNet [18] trials, where children from 3 years of age were treated with a daily dose of 7.5 mg of oral insulin. It is possible that our finding was due to the exposure of younger children or the use of higher oral insulin doses that may increase the likelihood of skin exposure to study drug. The overall frequency of skin and subcutaneous tissue disorders among all reported adverse events (4.3%) is comparable to that in TrialNet (7.7%) [18]. All skin and subcutaneous tissue adverse events in our study were classified as mild, resolved during the course of the study and were not correlated with other blood chemistry measurements or inflammatory markers.

In addition to establishing safety, our objective was to find evidence for a treatment-induced immune response. The study design and sample size were based on results from the previous Pre-POInT study, which enrolled children at 2–7 years of age with greater genetic risk [24]. Using the same outcomes and measurement methods, we observed a higher overall reactivity to insulin in the placebo group in this study (67%) than in the previous Pre-POInT study (20%), markedly reducing the study power. The younger age of the children is a major difference of the current study and is likely to contribute to the higher observed frequency of immune responses to insulin in the placebo group. Evidence for this includes the association between the antibody responses to insulin and younger age, and the correlation between age and T cell and monocyte subset compositions, with the latter being associated with the T cell responses to insulin.

Limitations of the study include our misjudgement on the effect size in favour of oral insulin, leading to the inclusion of 44 children, and the short follow-up period on relatively few children, which prevented us from assessing the efficacy of treatment in preventing islet autoimmunity or type 1 diabetes. All participants were of European extraction and the study cannot assess effects in other racial groups. A strength of the study is that, despite the challenge of obtaining blood samples from young children, adherence to the study protocol was high with comprehensive sample and data collection. These data included deep phenotyping of the immune responses and microbiome during early childhood and provided insights into how oral insulin might perturb the immune system and into disease mechanism. As there were no previous data to justify their inclusion in primary analyses, a number of these findings were based on exploratory and post hoc analyses, and, therefore, require validation in subsequent studies such as the POInT trial [37].

A potentially important finding was that the *INS* genotype appeared to influence antibody responses to treatment. In particular, we observed an association between oral insulin and the antibody response in children with the susceptible *INS* AA genotype, suggesting that the *INS* gene may modify the likelihood of the immune system responding to oral insulin. The response was observed by 6 months of treatment, corresponding to a 22.5 mg dose, which is lower than in the previous study [24] and may reflect the lower body weight of children in the present study. Genetic susceptibility criteria in the previous study were more stringent and it is likely that the majority of participants had the susceptible AA genotype. We also discovered differences in the microbiome composition between children with susceptible and non-susceptible *INS* genotypes and also minor effects after oral insulin treatment in children with the susceptible *INS* AA genotype. This included differences in bacterial diversity and richness, and an increased abundance of *Bacteroides dorei* in children with the susceptible *INS* genotype, a finding that is consistent with the increased abundance of *Bacteroides dorei* in children who developed type 1 diabetes in a Finnish study [38]. Unlike the antibody outcome, the in vitro T cell responses to insulin were not associated with treatment after stratification by the *INS* genotype. The T cell responses were, however, strongly associated with monocyte CD169 expression, providing new insights into disease pathogenesis. Monocyte CD169 expression is a sensitive marker of a type 1 interferon signature, which increases before islet autoantibody seroconversion in young children and is associated with respiratory infection [36]. CD169^+^ samples were surprisingly frequent and observed in the majority of children. They were also associated with recent adverse events, younger age and several other inflammatory markers. Early infection is associated with islet autoimmunity [39–41] and type 1 diabetes [42]. Thus, our findings that in vitro T cell responses to insulin were more likely to occur in CD169^+^ samples in children with the susceptible *INS* AA genotype may be relevant to the mechanism of insulin autoimmunity. We believe that our results do not reflect the presence of in vivo-primed T cells, but rather a heightened ability of the CD169^+^ monocytes to activate naive T cells in the in vitro assay. Children with the susceptible *INS* genotype are expected to have more peripheral insulin-autoreactive T cells [13, 14]. Extending our in vitro findings, we suggest that a type 1 interferon response to infection in antigen-presenting cells in vivo further increases the likelihood of activating these T cells and eventually leads to insulin autoimmunity. The observation that the insulin-responsive cells from the CD169^+^ samples contained more Th1/Th21 cells and fewer Tregs supports this hypothesis, and may also explain our previous finding of proinflammatory, proinsulin-responsive T cells in infants who later developed islet autoimmunity [25].

Overall, this study demonstrated safety for high-dose oral insulin administration in young children. The study did not reach its primary outcome of immune efficacy. Exploratory analyses, however, provided evidence of an interaction between an immune response to treatment and the *INS* gene as previously demonstrated for autoantigen. We, therefore, advocate that ongoing trials that include insulin or peptides of proinsulin as antigen-specific immunotherapy [43, 44] should incorporate stratification by *INS* genotype into their study design and analyses.

## Supplementary Information

ESM 1 (PDF 1.72 mb)

## Data Availability

All reasonable requests for raw and analysed data and materials will be promptly reviewed by the corresponding author to determine whether the request is subject to confidentiality obligations. Any data and materials that can be shared will be available from the corresponding author on reasonable request, with appropriate additional ethical approvals, and released via a material transfer agreement.

## Abbreviations

DPT-1: Diabetes Prevention Trial–Type 1
GADA: Autoantibodies to GAD
IAA: Autoantibodies to insulin
IA-2A: Autoantibodies to insulinoma-associated antigen-2
OTU: Operational taxonomic unit
POInT: Primary Oral Insulin Trial
Pre-POInT: Pre-Primary Oral Insulin Trial
SI: Stimulation index
ZnT8A: Autoantibodies to zinc transporter-8
