# Supplementary material for: Oral insulin immunotherapy in children at risk for type 1 diabetes in a randomised controlled trial
Source: Diabetologia. 2021 Jan 30;64(5):1079–92. doi: 10.1007/s00125-020-05376-1 (PMC8012335; doi:10.1007/s00125-020-05376-1)
Supplement: Supplementary file 1 — (PDF 1.72 mb) [file 125_2020_5376_MOESM1_ESM.pdf]

Electronic supplementary material (ESM)

Oral insulin immunotherapy in children at risk for type 1 diabetes in a randomised controlled trial

Robin Assfalg[1,2,3], Jan Knoop[1], Kristi L. Hoffman[4], Markus Pfirrmann[5], Jose Maria Zapardiel-Gonzalo[1], Anna Hofelich[2], Anne Eugster[6,7], Marc Weigelt[6], Claudia Matzke[1], Julia Reinhardt[6], Yannick Fuchs[6], Melanie Bunk[1], Andreas Weiss[1], Markus Hippich[1], Kathrin Halfter[5], Stefanie M. Hauck[8], Jörg Hasford[5], Joseph F. Petrosino[4], Peter Achenbach[1,2,3], Ezio Bonifacio[3,6,7,9], Anette-Gabriele Ziegler[1,2,3]

[1]Institute of Diabetes Research, Helmholtz Zentrum München, German Research Center for Environmental Health, Munich-Neuherberg, Germany

[2]Forschergruppe Diabetes, Technical University Munich, at Klinikum rechts der Isar, Munich, Germany

[3]German Center for Diabetes Research (DZD), Munich, Germany

[4]Alkek Center for Metagenomics and Microbiome Research, Department of Molecular Virology and Microbiology, Baylor College of Medicine, Houston, TX, USA

[5]Institute for Medical Information Processing, Biometry, and Epidemiology, Ludwig-Maximilians-University Munich, Munich, Germany

[6]Technische Universität Dresden, Center for Regenerative Therapies Dresden, Dresden, Germany

[7]Paul Langerhans Institute Dresden of the Helmholtz Center Munich at University Hospital Carl Gustav Carus and Faculty of Medicine, TU Dresden, Dresden, Germany

[8]Research Unit Protein Science, Helmholtz Zentrum München, German Research Center for Environmental Health, Munich-Neuherberg, Germany

[9]Institute for Diabetes and Obesity, Helmholtz Diabetes Center at Helmholtz Zentrum München, Munich-Neuherberg, Germany

Robin Assfalg, Jan Knoop and Kristi L. Hoffman are joint first authors.

Corresponding author
Anette-Gabriele Ziegler,
E-mail: anette-g.ziegler@helmholtz-muenchen.de

Table of content

ESM Methods....4

Randomisation and masking....4

Investigational Medicinal Product....4

Measurements of islet autoantibodies....4

Measurements of immune responses to insulin....4

Phenotyping of lymphocytes and monocytes....5

Single-cell gene expression profiling of CD4+ T cells responding to insulin....5

Blood cell counts, blood chemistry, electrolytes, IgE....6

Plasma inflammatory markers....6

Stool microbiome....6

INS genotyping....7

Recording of adverse events....7

Statistical comparisons....7

References....8

ESM Results....9

IgE....9

Laboratory analysis....9

Adverse events....9

Protocol violations....9

Tables

ESM Table 1 | Treatment duration and adherence....10

ESM Table 2 | Blood glucose, insulin and c-peptide measurements....11

ESM Table 3a | Full blood examination....12

ESM Table 3b | Blood chemistry....13

ESM Table 4a | Adverse advents (AEs) and severity grade....14

ESM Table 4b | Adverse events of the system organ class “skin....15

ESM Table 5 | Maximum antibody and CD4+ T cell response to insulin (*INS AA* genotype)....16

ESM Table 6 | Correlations between cell frequencies, CD169+ monocytes, inflammatory markers, and age....17

ESM Table 7 | List of genes and primers used for single cell gene expression profiling....20

Figures

ESM Fig. 1 | Flow analysis scheme to identify insulin responsive T cells....23

ESM Fig. 2 | Skin or subcutaneous tissue disorders....24

ESM Fig. 3 | Microbiome alterations in relation to age by WGS....25

ESM Fig. 4 | Relative abundance of species level taxonomies in relation to age....26

ESM Fig. 5 | Microbiome alterations in relation to age, treatment, and genotype....27

ESM Fig. 6 | Immune cell population frequencies over time....28

ESM Fig. 7 | Immune profiling markers in the study participants....29

ESM Fig. 8 | Monocyte CD169 expression associations with microbiome and age....30

ESM Fig. 9 | $CD8^+$ T cell responses to insulin (SI)....31

ESM Fig. 10 | Gene expression in insulin-responsive $CD4^+$ T cells....32

ESM Methods

Randomisation and masking

A computer-generated randomisation list was prepared with an allocation ratio of 1:1 (placebo to oral insulin) using a web-based system (https://wwwapp.ibe.med.uni-muenchen.de/randoulette/). All investigators and participants were masked to the treatment allocation. Unblinding was not necessary during the study.

Investigational Medicinal Product

Insulin crystals were provided by Lilly Pharmaceuticals (Indianapolis, Indiana). The investigational medicinal products (insulin and placebo) were manufactured as identical capsules containing either insulin crystals (7.5 mg, 22.5 mg, or 67.5 mg) in microcrystalline cellulose (total capsule content 200 mg) or 200 mg microcrystalline cellulose placebo by InPhaSol, Apotheke des Universitätsklinikums Heidelberg, Germany. The drug packages were sequentially numbered according to the randomly allocated treatment. Parents were instructed to sprinkle the contents of one capsule onto one teaspoon of food (e.g. yogurt, breast milk, or commercial baby food) for administration once daily.

Measurements of islet autoantibodies

Autoantibodies to glutamic acid decarboxylase (GADA), insulinoma-associated antigen 2 (IA-2A), and zinc transporter-8 (ZnT8A) were measured using harmonized radio binding methods [1, 2]. Islet autoantibody assays were evaluated according to the Diabetes Antibody Standardisation Program [3-5].

Measurements of immune responses to insulin

Insulin autoantibody (IAA) levels were measured using a competitive radio binding assay [6, 7]. A positive response was defined as a value of ≥1.5 and a ≥2-fold increase from baseline. Serum IgG binding to insulin was measured by a non-competitive radio binding assay with protein-G capture of IgG [8]. A positive response was defined as an increase of >10 counts/min from the baseline value. Salivary IgA binding to insulin was measured using a radio binding assay as previously described [9]. A positive response was defined as an increase of ≥ 3-fold from baseline. Serum IgE against insulin was measured using a radio binding assay [10].

$CD4^+$ T cell antigen responses were measured using stored frozen peripheral blood mononuclear cells (PBMCs). Responses were measured using a dye (Cell Proliferation Dye eFluor 670, eBioscience, San Diego, CAL, USA) dilution assay, quantifying proliferation (eFluor670dim cells) and activation ($CD25^+$) after 5 days of culture without or with the antigen insulin that was identical to the insulin administered to the children (50 µg/ml, Lilly Pharmaceuticals) as previously described [9] (ESM Fig. 1). The assay included a median of 12 wells containing 200,000 eFlour670 dye-labelled cells in medium plus insulin and 6 wells with cells and medium alone. The SI was calculated as the number of $CD4^+$ eFluor670dim$CD25^+$ cells per 50,000 acquired live $CD4^+$ T cells in all wells containing insulin relative to the number of $CD4^+$ eFluor670dim$CD25^+$ cells per 50,000 acquired live $CD4^+$ T cells in all wells containing medium alone. A positive sample was defined as an SI of >3. A positive T cell outcome was defined as a positive sample and an increase in SI of >2-fold at any follow-up visit relative to the

baseline value. $CD8^+$ T cell proliferation responses to insulin were also measured in the same assay by gating on $CD8^+CD4^-$ T cells (ESM Fig. 1).

Phenotyping of lymphocytes and monocytes

Freshly isolated PBMCs (2.5×10^5 cells) were incubated for 1 min at room temperature with Fc receptor blocking reagent (Miltenyi Biotec, Bergisch Gladbach, Germany). Cell surface markers were stained for 20 min at 4 °C in phosphate-buffered saline (PBS) without $Ca2^+$ and $Mg2^+$ (PBS−/−; Gibco) containing 0.5% bovine serum albumin using the following mouse anti-human monoclonal antibodies: anti-CD3 Alexa Fluor 700 (clone HIT3α; BioLegend, San Diego, CAL, USA), anti-CD4 Brilliant Violet 510 (SK3; BD Biosciences, San Jose, CAL, USA), anti-CD8a Brilliant Violet 605 (RPA-T8), anti-CD14 Pacific Blue (HCD14), anti-CD16 PerCP-Cy5.5 (3G8; all BioLegend, San Diego, CAL, USA), anti-CD25 PE (M-A251; BD Biosciences, San Jose, CAL, USA), anti-CD45RA PE-Cy5 (HI100; BioLegend, San Diego, CAL, USA), anti-CD69 fluorescein isothiocyanate (FN50; BD Biosciences, San Jose, CAL, USA), anti-CD127 PE-Cy7 (A019D5) and anti-CD169 Alexa Fluor 647 (7-239; both BioLegend, San Diego, CAL, USA). Cells were washed twice in PBS−/− and stained for 20 min at room temperature with Zombie NIR (BioLegend, San Diego, CAL, USA) to evaluate cell viability. PBMCs were fixed with 1.5% formalin in PBS−/− and analysed within 24 h on a flow cytometer (LSR Fortessa, Becton Dickinson, Franklin Lakes, NJ, USA) using FACSDiva acquisition software (Version 7.0; BD Biosciences, San Jose, CAL, USA). FlowJo software (Version 10; TreeStar Inc., Ashland, OR, USA) was used to analyse lymphocyte and monocyte subsets.

Single-cell gene expression profiling of $CD4^+$ T cells responding to insulin

$CD4^+$ T cells that had proliferated, as determined by eFluor® 670 dilution, and displayed CD25 upregulation were identified as responding cells and were single-cell-sorted directly into 96-well microplates containing 5 µl of PBS prepared with diethylpyrocarbonate-treated water. For samples that had an SI against insulin above 3, cells were processed for gene expression. cDNA was synthesized directly from cells using qScript™ cDNA Supermix (Quanta Biosciences, Gaithersburg, MD, USA). Total cDNA was pre-amplified for 18 cycles, with 1 cycle of denaturation at 95 °C for 1 min, followed by cycling at 95 °C for 15 s, 60 °C for 1 min, and 72 °C for 1.5 min, followed by one cycle of 72 °C for 7 min, with TATAA GrandMaster Mix (TATAA Biocenter, Göteborg, Sweden) in the presence of 76 primer pairs at a final volume of 35 µl (ESM Table 7). Then, 10 µl of preamplified DNA was treated with 1.2 units of exonuclease I. To quantify gene expression, real-time PCR was performed on the BioMark™ HD System (Fluidigm Corporation, South San Francisco, CA, USA) using the 96.96 Dynamic Array IFC according to the GE 96 × 96 Fast PCR+ Melt protocol with SsoFast EvaGreen Supermix containing Low ROX (Bio-Rad) and 5 µM of primers in each assay. The primers and target genes are listed in Table S7. Raw data were analyzed using Fluidigm Real-Time PCR analysis software and GenEx Pro 5.3.6 Software (MultiD, Göteborg, Sweden). Additional data analysis was done using KNIME 2.5.2 software (KNIME AG, Zürich, Switzerland). Analysis of multivariate gene expression patterns was performed by Uniform Manifold Approximation and Projection for Dimension Reduction (UMAP) [11] and unsupervised WARD hierarchical clustering (hclust) on the pre-processed Ct values. For pre-processing, a linear model was used to correct for

potential confounding effects, which can mask relevant biological variability [12]. In brief, batch effects (dummy coding for each plate/batch) were modelled jointly with dose effects by regressing out the effect of plates on each individual gene while controlling for dose in order to obtain a corrected gene expression dataset.

Blood glucose, insulin, and C-peptide

Plasma glucose was measured by an accredited laboratory (Medizet, Städtisches Klinikum München GmbH, Munich, Germany). Serum insulin and C-peptide concentrations were measured by fluorescence enzyme immunoassays using an automated immunoassay analyser (AIA-360, Tosoh Bioscience Inc., South San Francisco, CA, USA).

Blood cell counts, blood chemistry, electrolytes, IgE

Blood cell counts, GOT, GPT, GGT, AP, albumin, creatinine, sodium, potassium, and IgE concentrations were measured by an accredited laboratory (Medizet, Städtisches Klinikum München GmbH, Munich, Germany).

Plasma inflammatory markers

Inflammation-related protein biomarkers were determined after unblinding of participants. Measurements were performed by proximity extension assay using the Olink inflammation panel (Olink, Uppsala, Sweden) following manufacturer's instructions.

Stool microbiome

Stool samples were collected at home at the day of the visit or within two days before or after the visit at baseline, 6 months, and 12 months, into tubes containing ethanol that were provided to the parents. The samples were brought to the visit, aliquoted, and stored at −80 °C. Alternatively, samples could be sent to the central laboratory with guaranteed delivery within 24 h. The bacterial component of the microbiome in each stool sample was analyzed by 16S rRNA gene compositional analysis, as previously described [13]. To generate 16S rDNA data, genomic bacterial DNA was extracted from the samples using MO BIO PowerMag Soil DNA Isolation Kit (Qiagen, Venlo, Netherlands). The 16S rDNA V4 region was amplified by PCR and sequenced in the MiSeq platform (Illumina, San Diego, CA, USA) using the 2 × 250 bp paired-end protocol. The primers used for amplification contained adapters for MiSeq sequencing and dual-index barcodes so that the PCR products may be pooled and sequenced directly [14], targeting at least 10,000 reads per sample. The standard pipeline for processing and analysing the 16S rDNA gene data incorporated phylogenetic and alignment-based approaches to maximize data resolution. The read pairs were demultiplexed based on the unique molecular barcodes, and reads were merged using USEARCH v7.0.1001 [15]. 16S rRNA gene sequences were assigned into Operational Taxonomic Units (OTUs) or phylotypes at a similarity cut off value of 97% using the UPARSE pipeline. Abundances were recovered by mapping the demultiplexed reads to the UPARSE OTUs.

A subset of samples with sufficient material was selected for metagenomic whole genome shotgun (WGS) sequencing for deeper characterisation. Metagenomic WGS sequencing utilized the same extracted bacterial genomic DNA used for 16S rDNA compositional analysis. For WGS, individual libraries constructed from each

sample were loaded into the HiSeq platform (Illumina, San Diego, CA, USA) and sequenced using the 2x100 bp pair-end read protocol. The process of quality filtering, trimming and demultiplexing was carried out by in-house pipeline developed by assembling a number of publicly available tools such as Casava v1.8.3 (Illumina, San Diego, CA, USA) for the generation of fastqs, Trim Galore and cut adapt for adapter and quality trimming, and PRINSEQ for sample demultiplexing. In addition, Bowtie2 v2.2.1 [16] was used to map reads to custom databases for bacteria, viruses, human, and vectors. Reads whose highest identity match was not bacterial were removed from subsequent analysis. For bacterial reads, the highest identity match was chosen. If there were multiple top hits, the lowest common ancestor was determined.

INS genotyping

Genomic DNA was amplified using primers (forward: 5'-GGTCTGTTCCAAGGGCCTTT-3'; biotinylated reverse: 5'-ATGGCAGAAGGACAGTGATCTGG-3') targeting rs689 of *INS* and the SsoFast EvaGreen Supermix on a CFX96 system (Bio-Rad, Hercules, CA; USA) according to manufacturer's protocol. Subsequently, genotyping of rs689 was performed by pyrosequencing on a PyroMark Q48 Autoprep using a sequencing primer (5'-CTCAGCCCTGCCTGT-3') and PyroMark Q48 Advanced Reagents (Qiagen, Venlo, Netherlands) according to manufacturer's protocol. Primer design and SNP analysis was carried out using the PyroMark Assay Design 2.0 and PyroMark Q48 Autoprep 2.4.2 Software (Qiagen, Venlo, Netherlands), respectively.

Recording of adverse events

Throughout the study, the investigators recorded any adverse events using an adverse event clinical report form, regardless of the event's severity or relation to the study drug or study procedure. The families were instructed to note any symptoms of hypoglycaemic events such as trembling, sweating or impaired consciousness after study drug intake. Hypoglycaemia was defined as a blood glucose level <2.78 mmol/l (<50 mg/dl).

Statistical comparisons

Additional analyses were planned to compare the immunological outcomes in children with the *INS AA* genotype and treatment effects on the stool microbiome. These and all other analyses were considered exploratory. An interaction between *INS* genotype and treatment on immunological responses to insulin was assessed using the Cox proportional hazards model. All analyses comparing responses in relation to monocyte CD169 expression, and analyses of cell frequency and plasma inflammatory markers were defined post-hoc. Spearman's correlation was calculated to assess the correlation between two continuous variables. Differences between groups' centroids defined by a principal component analysis (PCA) were assessed using a permutational multivariate analysis of variance (PERMANOVA). Analysis of age relationships to cell population frequencies included a linear mixed model with the cell frequencies as fixed effects and the children identification numbers as a random effect was fitted to predict the age. Stool analyses were conducted to characterize differences in the microbiome between the two treatment groups, including stratification by *INS AA* genotype and to determine the relationship of the microbiome to the immune responses in blood. Differences in beta diversity were visualized using a principal coordinates analysis (PCoA) followed by a PERMANOVA to assess differences between groups.

References

[1] Bonifacio E, Yu L, Williams AK, et al. (2010) Harmonization of glutamic acid decarboxylase and islet antigen-2 autoantibody assays for national institute of diabetes and digestive and kidney diseases consortia. The Journal of clinical endocrinology and metabolism 95: 3360-3367

[2] Achenbach P, Lampasona V, Landherr U, et al. (2009) Autoantibodies to zinc transporter 8 and SLC30A8 genotype stratify type 1 diabetes risk. Diabetologia 52: 1881-1888

[3] Törn C, Mueller PW, Schlosser M, Bonifacio E, Bingley PJ, Participating Laboratories (2008) Diabetes antibody standardization program: evaluation of assays for autoantibodies to glutamic acid decarboxylase and islet antigen-2. Diabetologia 51: 846–852

[4] Lampasona V, Schlosser M, Mueller PW et al. (2011) Diabetes antibody standardization program: first proficiency evaluation of assays for autoantibodies to zinc transporter 8. Clin Chem 57:1693–1702

[5] Schlosser M, Mueller PW, Törn C, Bonifacio E, Bingley PJ, Participating Laboratories (2010) Diabetes antibody standardization program: evaluation of assays for insulin autoantibodies. Diabetologia 53: 2611–2620

[6] Naserke HE, Dozio N, Ziegler AG, Bonifacio E (1998) Comparison of a novel micro-assay for insulin autoantibodies with the conventional radiobinding assay. Diabetologia 41: 681-683

[7] Williams AJ, Bingley PJ, Bonifacio E, Palmer JP, Gale EA (1997) A novel micro-assay for insulin autoantibodies. Journal of autoimmunity 10: 473-478

[8] Koczwara K, Muller D, Achenbach P, Ziegler AG, Bonifacio E (2007) Identification of insulin autoantibodies of IgA isotype that preferentially target non-human insulin. Clinical immunology 124: 77-82

[9] Bonifacio E, Ziegler AG, Klingensmith G, et al. (2015) Effects of high-dose oral insulin on immune responses in children at high risk for type 1 diabetes: the Pre-POINT randomized clinical trial. Jama 313: 1541-1549

[10] Bonifacio E, Scirpoli M, Kredel K, Fuchtenbusch M, Ziegler AG (1999) Early autoantibody responses in prediabetes are IgG1 dominated and suggest antigen-specific regulation. Journal of immunology 163: 525-532

[11] Becht E, McInnes L, Healy J, et al. (2018) Dimensionality reduction for visualizing single-cell data using UMAP. Nat Biotechnol

[12] Buettner F, Natarajan KN, Casale FP, et al. (2015) Computational analysis of cell-to-cell heterogeneity in single-cell RNA-sequencing data reveals hidden subpopulations of cells. Nat Biotechnol 33: 155-160

[13] Stewart CJ, Ajami NJ, O'Brien JL, et al. (2018) Temporal development of the gut microbiome in early childhood from the TEDDY study. Nature 562: 583-588

isotype that preferentially target non-human insulin. Clinical immunology 124: 77-82

[14] Caporaso JG, Lauber CL, Walters WA, et al. (2012) Ultra-high-throughput microbial community analysis on the Illumina HiSeq and MiSeq platforms. ISME J 6: 1621-1624

[15] Edgar RC (2010) Search and clustering orders of magnitude faster than BLAST. Bioinformatics 26: 2460-2461

[16] Langmead B, Salzberg SL (2012) Fast gapped-read alignment with Bowtie 2. Nat Methods 9: 357-359

ESM Results

IgE

IgE concentrations were above the reference limits in 3 children at the 12 months visit; all three children were in the placebo group. Children in the group receiving oral insulin showed no change in measured IgE (Median difference = 0.00). No child had IgE to insulin.

Laboratory analysis

No significant changes in blood cell counts were observed. Monocyte count at baseline was the only parameter with values that were significantly different between the children in the placebo group and the oral insulin group (median 7% versus 5%; $p = 0.0089$, ESM Table 3a). No differences in blood chemistry values were observed except for GGT at 12 months with higher values in children in the placebo group (median 11 U/l vs 9 U/l, $p = 0.0063$, ESM Table 3b).

Adverse events

A total of 114 adverse events were reported over a cumulative exposure period of 21.1 years in 21/21 children in the placebo group (5.64 events per year), and 181 adverse events were reported over a cumulative exposure period of 21.7 years in 22/22 children in the oral insulin group (8.38 events per year). The time to the first observed adverse event was similar between the two groups ($p = 0.39$; log rank test). Infections were the most frequently reported adverse events (ESM Table 4). The severity of adverse events was similar between the two groups. There were six serious adverse events, four in the oral insulin group and two in the placebo group, none of which were considered related to the study drug. By system organ class, the frequency of skin and subcutaneous tissue disorders was greater in the oral insulin group (12 events in 8 children) than in the placebo group (1 event in 1 child; $p = 0.01$; ESM Fig. 2). These included diaper rash, erythema, eczema, pruritus, and urticaria (ESM Table 4). The overall frequency of skin and subcutaneous tissue disorders among all reported adverse events was 4.3% and all of these adverse events were classified as mild (grade 1) and resolved.

Protocol violations

There were 332 protocol deviations, all except one classified as minor. Most protocol violations were associated with missing values regarding single blood count parameters, missing parameters during blood glucose monitoring, missing parameters during the physical examination or an exceeded time window for study visits. The one protocol violation judged as major was the dispensation of a wrong medication package to one subject. However, after unblinding it turned out that this wrongly dispensed medication package was from the same medication group (placebo) the child had received in the trial.

ESM Table 1 | Treatment duration and adherence

	Placebo	Oral insulin 7.5 mg	Oral insulin 22.5 mg	Oral insulin 67.5 mg
Treatment duration per participant, median (IQR), months	11.95 (11.7 - 12.2)	3.00 (2.9 - 3.3)	3.00 (2.9 - 3.2)	6.00 (5.8 - 6.2)
Treatment duration cumulative; months	252.5	66.7	67.2	126.2
Family reported adherence to medication, median (IQR); %	97.95 (94.2 - 99.0)	98.85 (96.3 - 100.0)	97.65 (94.1 - 98.9)	95.00 (89.0 - 98.7)

ESM Table 2 | Blood glucose, insulin and c-peptide measurements

Visit	Treatment	Time point (minutes)	Glucose (mmol/l) median (IQR)	AUC glucose median (IQR)	Insulin (pmol/l) median (IQR)	AUC insulin median (IQR)	C-peptide (nmol/l) median (IQR)	AUC c-peptide median (IQR)
1	Placebo	-10	4.7 (4.3-5.1)	10710 (10380-11640)	36.1 (16.7-63.2)	0.17 (0.12-0.33)	0.56 (0.33-1.06)	1.73 (1.46-3.74)
		30	5.3 (4.7-5.9)		56.3 (42.7-123.6)		0.73 (0.46-1.16)	
		60	5.2 (4.8-5.3)		59.7 (26.4-120.0)		0.58 (0.43-1.21)	
		120	4.9 (4.7-5.4)		49.7 (29.7-114.2)		0.53 (0.43-1.09)	
	Oral insulin 7.5mg	-10	4.9 (4.4-5.4)	11205 (10635-11730)	24.3 (5.6-52.8)	0.11 (0.04-0.19)	0.46 (0.20-0.63)	1.58 (0.92-2.06)
		30	5.3 (4.7-6.2)		83.7 (26.2-163.2)		0.75 (0.52-1.07)	
		60	5.1 (4.8-5.4)		50.0 (19.8-91.3)		0.73 (0.51-0.89)	
		120	5.1 (4.7-5.4)		37.2 (20.8-65.6)		0.60 (0.46-0.75)	
2	Placebo	-10	4.5 (4.1-5.0)	11130 (9945-11520)	18.1 (8.3-45.8)	0.20 (0.10-0.40)	0.57 (0.27-0.74)	1.51 (0.74-2.65)
		30	5.0 (4.7-5.7)		49.3 (30.2-111.5)		0.73 (0.41-1.24)	
		60	4.9 (4.7-5.4)		55.6 (22.2-153.5)		0.66 (0.46-1.26)	
		120	5.1 (4.6-5.3)		70.5 (52.3-130.6)		0.78 (0.62-1.00)	
	Oral insulin 22.5mg	-10	4.6 (4.2-4.9)	10785 (9915-11295)	38.2 (17.2-68.4)	0.15 (0.10-0.26)	0.46 (0.30-0.79)	1.45 (0.84-2.05)
		30	5.1 (4.5-5.8)		101.0 (49.3-195)		0.75 (0.50-1.26)	
		60	5.1 (4.6-5.4)		75.7 (34.0-118.8)		0.73 (0.40-1.05)	
		120	4.9 (4.6-5.4)		55.6 (33.5-132.5)		0.60 (0.50-1.07)	
3	Placebo	-10	4.6 (4.3-5.1)	11115 (10613-12360)	26.4 (12.2-64.1)	0.23 (0.15-0.37)	0.46 (0.38-0.90)	1.64 (0.94-2.11)
		30	5.5 (4.7-6.4)		119.8 (59.7-198)		0.93 (0.60-1.39)	
		60	5.1 (4.7-5.6)		88.2 (36.3-133.9)		0.83 (0.50-1.18)	
		120	5.2 (4.6-5.6)		56.3 (34.0-112.8)		0.60 (0.43-0.96)	
	Oral insulin 67.5mg	-10	4.6 (4.2-4.7)	10718 (9840-11310)	42.4 (23.3-106.9)	0.23 (0.16-0.23)	0.53 (0.43-0.98)	2.07 (1.33-2.77)
		30	5.0 (4.4-5.8)		93.4 (63.2-153.6)		0.85 (0.61-1.17)	
		60	4.8 (4.4-5.3)		95.8 (35.4-118.4)		0.96 (0.52-1.06)	
		120	5.2 (4.9-5.4)		66.7 (49.7-97.6)		0.79 (0.47-0.98)	

ESM Table 3a | Full blood examination

Parameter	Unit	Visit	Placebo median (IQR)	Oral insulin median (IQR)
RBC	10^{12}/l	1	4800 (4600-5000)	4600 (4400-4800)
		5	4800 (4700-4900)	4700 (4500-4800)
Haemoglobin	g/l	1	121.0 (118.0-129.0)	120.0 (117.0-122.0)
		5	123.0 (119.0-125.0)	122.0 (119.0-125.0)
MCHC	g/l	1	340.0 (335.0-345.0)	339.0 (334.0-350.0)
		5	34.1 (338.0-346.0)	34.5 (339-349)
MCH	pg	1	25.9 (24.4-26.8)	26.2 (25.2-27.3)
		5	25.6 (24.9-26.6)	25.6 (25.2-26.2)
MCV	fl	1	76.3 (72.8-78.0)	76.8 (73.9-79.8)
		5	75.3 (72.8-77.0)	74.2 (72.8-77.2)
Haematocrit	%	1	0.36 (0.38-0.38)	0.35 (0.35-0.37)
		5	0.36 (0.35-0.37)	0.35 (0.34-0.37)
WBC	10^9/l	1	9.6 (8.6-11.6)	9.0 (7.4-11.0)
		5	8.2 (6.2-9.7)	8.3 (6.6-10.3)
Platelets	10^9/l	1	397.0 (332.0-462.0)	340.0 (310.0-444.0)
		5	354.0 (325.0-430.0)	336.0 (277.0-404.0)
Eosinophils	%	1	0.04 (0.01-0.06)	0.03 (0.02-0.05)
		5	0.03 (0.01-0.04)	0.04 (0.02-0.07)
Basophils	%	1	0.001 (0.001-0.01)	0.001 (0.001-0.01)
		5	0.01 (0.001-0.01)	0.01 (0.001-0.01)
Lymphocytes	%	1	0.51 (0.40-0.54)	0.56 (0.49-0.62)
		5	0.49 (0.39-0.58)	0.51 (0.44-0.59)
Monocytes*	%	1	0.07 (0.06-0.10)*	0.05 (0.02-0.06)[a]
		5	0.08 (0.06-0.10)	0.06 (0.05-0.07)

[a] Monocyte count at baseline is significantly different between the two groups; $p = 0.0089$, Mann-Whitney U Test

ESM Table 3b | Blood chemistry

Parameter	Unit	Visit	Placebo median (IQR)	Oral insulin median (IQR)
Sodium	mmol/l	1	137.5 (137.0-139.0)	138.0 (136.0-138.0)
		5	140.0 (139.0-141.0)	140.0 (138.0-141.0)
Potassium	mmol/l	1	4.4 (4.0-4.8)	4.6 (4.0-4.9)
		5	4.4 (4.3-4.6)	4.4 (4.2-4.4)
GOT	µkat/l	1	0.74 (0.65-0.83)	0.76 (0.67-0.90)
		5	0.73 (0.65-0.85)	0.72 (0.65-0.78)
GPT	µkat/l	1	0.32 (0.30-0.43)	0.35 (0.30-25.0)
		5	0.33 (0.30-0.42)	0.33 (0.28-0.33)
GGT	µkat/l	1	0.18 (0.15-0.20)	0.15 (0.13-0.18)
		5	11.0 (0.15-0.22)	0.15 (0.13-0.17)[a]
AP	µkat/l	1	4.39 (3.86-5.79)	4.17 (3.81-4.54)
		5	4.76 (4.07-5.66)	4.02 (3.52-4.94)
Albumin	g/l	1	41.0 (38.0-43.0)	41.0 (38.0-43.0)
		5	44.0 (43.0-46.0)	44.0 (43.0-46.0)
Creatinine	µmol/l	1	35.36 (26.52-35.36)	35.36 (26.52-35.36)
		5	26.52 (26.52-26.52)	26.52 (17.68-26.52)

[a] GGT at 12 months is significantly different between the two groups; *p* = 0.0063, Mann-Whitney U Test

ESM Table 4a | Adverse advents (AEs) and severity grade

	Placebo			Oral insulin			p-value
	Events (N=114)	Participants (N=21)		Events (N=181)	Participants (N=22)		
System Organ Class	n	n (%)		n	n (%)		
Blood and lymphatic system disorders	0	0	(0.0)	2	2	(9.1)	
Congenital. familial and genetic disorders	0	0	(0.0)	1	1	(4.5)	
Gastrointestinal disorders	12	8	(38.1)	30	13	(59.1)	0.22
General disorders	18	10	(47.6)	30	14	(63.6)	0.35
Immune system disorders	1	1	(4.8)	0	0	(0.0)	
Infections and infestations	72	17	(81.0)	77	20	(90.9)	0.54
Injury poisoning	0	0	(0.0)	4	4	(18.2)	
Investigations	1	1	(4.8)	1	1	(4.5)	
Metabolism and nutrition disorders	0	0	(0.0)	2	2	(9.1)	
Respiratory. thoracic disorders	8	5	(23.8)	20	8	(36.4)	0.17
Skin and subcutaneous tissue disorders	1	1	(4.8)	12	8	(36.4)	0.01
Surgical and medical procedures	1	1	(4.8)	2	2	(9.1)	
AE severity (grade)							
None reported		2	(9.5)		1	(4.6)	
Mild	109	16	(76.2)	170	15	(68.2)	
Moderate	3	2	(9.5)	9	5	(22.7)	
Severe and undesirable	2	1	(4.8)	2	1	(4.6)	
Life-threatening or disabling	0	0	(0.0)	0	0	(0.0)	
Death	0	0	(0.0)	0	0	(0.0)	

ESM Table 4b | Adverse events of the system organ class “skin and subcutaneous tissue disorders

Participant	Treatment	Event	Severity
1	Oral insulin	Pruritus	Mild
2	Oral insulin	Erythema	Mild
	Oral insulin	Erythema	Mild
3	Oral insulin	Exanthema	Mild
4	Placebo	Eczema	Mild
5	Oral insulin	Urticaria	Mild
	Oral insulin	Urticaria	Mild
6	Oral insulin	Diaper rash	Mild
7	Oral insulin	Diaper rash	Mild
	Oral insulin	Diaper rash	Mild
	Oral insulin	Diaper rash	Mild
8	Oral insulin	Eczema	Mild
9	Oral insulin	Eczema	Mild

ESM Table 5 | Maximum antibody and CD4$^+$ T cell response to insulin during treatment in participants carrying the *INS AA* genotype

Treatment group	IgG anti-insulin (max. increase from baseline)	Saliva IgA anti- insulin (max. fold over baseline)	IAA (max. units; fold over baseline*)	Antibody response	CD4$^+$ T cell response (max. SI; fold over baseline**)	T cell response
Placebo	7.8	1.1	0.8	No	5.24 ; **9.5**	**Yes**
Placebo	1.9	0.9	0.6	No	7.03 ; 1.1	No
Placebo	0.4	1.2	0.8	No	4.33 ; **8.9**	**Yes**
Placebo	4.4	1.2	1.1	No	1.54	No
Placebo	**27.9**	1.7	0.8	**Yes**	1.58	No
Placebo	3.4	1.4	0.9	No	3.65 ; **2.1**	**Yes**
Placebo	8.4	0.8	0.7	No	3.06 ; **3.2**	**Yes**
Placebo	8.1	1.3	0.5	No	1.01	No
Placebo	4.2	1.8	0.6	No	1.61	No
Placebo	**23.2**	1.2	0.3	**Yes**	1.79	No
Placebo	3.2	1.4	0.7	No	3.10 ; **4.1**	**Yes**
Insulin	6.2	1.2	0.9	No	1.98	No
Insulin	**16.2**	**5.9**	0.6	**Yes**	14.16 ; **10.5**	**Yes**
Insulin	**23.4**	1.3	1.0	**Yes**	2.92	No
Insulin	0.4	0.9	2.8 ; **55.8**	**Yes**	1.67	No
Insulin	**10.1**	n.a.	0.7	**Yes**	1.45	No
Insulin	**13.1**	1.5	0.9	**Yes**	3.44 ; **2.6**	**Yes**
Insulin	**10.3**	1.2	1.0	**Yes**	6.16 ; **3.6**	**Yes**
Insulin	8.2	**5.0**	2.2 ; 1.1	**Yes**	1.09	No
Insulin	**19.5**	1.5	0.6	**Yes**	0.82	No
Insulin	3.1	0.9	0.4	No	3.95 ; **4.9**	**Yes**
Insulin	6.5	1.9	0.3	No	1.59	No

*Fold over baseline is shown if IAA response >1.5 units

**Fold over baseline is shown if CD4$^+$ T cell response is >3 SI

ESM Table 6 | Significant correlations between cell population frequencies, CD169^{+} monocytes, plasma inflammatory markers, and age

Variable 1	Variable 2	Spearman's Rho	p-value	Adjusted p-value
Memory CD4	Age	0.64	9.59E-25	1.36E-21
Memory Treg	Age	0.44	4.72E-11	6.52E-09
CD4	Age	-0.43	9.43E-11	1.16E-08
Memory CD8	Age	0.41	8.15E-10	8.25E-08
Age	IL.10RB	-0.60	1.59E-08	1.18E-06
Intermediate monocytes	CD169	0.36	1.25E-07	7.54E-06
Monocytes	Age	0.34	5.46E-07	2.69E-05
Age	CD5	-0.54	7.61E-07	3.66E-05
Memory CD4	DNER	-0.50	5.24E-06	2.01E-04
Age	FGF.23	-0.50	6.21E-06	2.30E-04
CD169	CXCL10	0.49	9.43E-06	3.22E-04
Activated CD4	CD169	0.30	9.79E-06	3.31E-04
Age	CCL4	-0.47	2.85E-05	8.21E-04
Age	FGF.21	-0.47	2.97E-05	8.41E-04
CD8	Age	0.28	4.29E-05	1.15E-03
Intermediate monocytes	IL10	0.45	4.77E-05	1.25E-03
Activated CD4	CXCL10	0.45	6.35E-05	1.61E-03
CD4	CD5	0.44	7.16E-05	1.78E-03
Memory CD8	DNER	-0.44	7.74E-05	1.90E-03
Activated CD8	TNF	0.44	8.78E-05	2.13E-03
Classical monocytes	Age	0.27	8.85E-05	2.14E-03
Monocytes	IL6	0.43	1.25E-04	2.77E-03
Activated CD8	CD169	0.26	1.28E-04	2.79E-03
Activated CD8	PD.L1	0.43	1.48E-04	3.09E-03
Non-classical monocytes	Age	-0.26	1.58E-04	3.28E-03
CD169	IL6	0.42	1.90E-04	3.78E-03
Memory Treg	CD5	-0.42	2.13E-04	4.08E-03
Memory CD4	FGF.23	-0.41	2.89E-04	5.27E-03
Age	CX3CL1	-0.41	2.90E-04	5.27E-03
CD169	CSF.1	0.41	3.04E-04	5.41E-03
Classical monocytes	FGF.5	0.41	3.23E-04	5.64E-03
CD8	MCP.1	-0.41	3.25E-04	5.64E-03
Non-classical monocytes	FGF.5	-0.41	3.31E-04	5.70E-03
Age	IL.17C	-0.41	3.37E-04	5.78E-03
Monocytes	DNER	-0.41	3.43E-04	5.86E-03
Memory CD4	CCL4	-0.40	3.51E-04	5.94E-03
Memory CD4	IL.10RB	-0.40	3.93E-04	6.50E-03
Activated CD8	IFN.gamma	0.40	4.03E-04	6.60E-03
Memory CD4	CD5	-0.40	4.27E-04	6.94E-03
CD3	CCL19	-0.39	5.70E-04	8.78E-03
Activated CD4	IFN.gamma	0.39	6.32E-04	9.35E-03
CD169	IFN.gamma	0.39	6.47E-04	9.54E-03
Monocytes	SCF	-0.38	7.36E-04	1.05E-02

Variable 1	Variable 2	Spearman's Rho	p-value	Adjusted p-value
Classical monocytes	OSM	-0.38	8.86E-04	1.20E-02
Age	MCP.3	-0.38	9.60E-04	1.28E-02
CD8	CD5	-0.37	9.97E-04	1.32E-02
Activated CD8	CXCL10	0.37	1.04E-03	1.36E-02
CD3	OPG	-0.37	1.06E-03	1.38E-02
CD169	MCP.2	0.37	1.13E-03	1.47E-02
CD4	MCP.1	0.37	1.17E-03	1.49E-02
Monocytes	CCL19	0.37	1.19E-03	1.51E-02
CD169	IL10	0.37	1.26E-03	1.56E-02
Non-classical monocytes	CCL4	0.37	1.29E-03	1.58E-02
CD8	DNER	-0.36	1.44E-03	1.71E-02
CD4	CX3CL1	0.36	1.71E-03	1.96E-02
Memory Treg	DNER	-0.36	1.77E-03	1.99E-02
Intermediate monocytes	CXCL10	0.36	1.79E-03	2.01E-02
CD4	DNER	0.36	1.85E-03	2.06E-02
Memory CD4	IL.17C	-0.35	2.09E-03	2.23E-02
Memory CD8	FGF.23	-0.35	2.31E-03	2.40E-02
CD169	CXCL11	0.35	2.34E-03	2.42E-02
CD169	LIF	0.35	2.42E-03	2.50E-02
Age	DNER	-0.35	2.48E-03	2.54E-02
Monocytes	4E.BP1	-0.34	2.71E-03	2.70E-02
Classical monocytes	CCL4	-0.34	2.81E-03	2.78E-02
Memory CD8	CX3CL1	-0.34	2.81E-03	2.78E-02
CD169	CDCP1	0.34	2.84E-03	2.80E-02
Treg	CCL19	0.34	2.91E-03	2.84E-02
Non-classical monocytes	OSM	0.34	2.95E-03	2.88E-02
Memory Treg	CD169	-0.20	3.15E-03	3.01E-02
Age	TGF.alpha	-0.34	3.29E-03	3.09E-02
Monocytes	PD.L1	0.34	3.47E-03	3.23E-02
CD169	PD.L1	0.33	3.78E-03	3.45E-02
Age	IL.12B	-0.33	3.81E-03	3.46E-02
Memory CD4	AXIN1	0.33	3.91E-03	3.52E-02
Memory Treg	AXIN1	0.33	4.22E-03	3.73E-02
Memory CD8	CXCL10	0.33	4.32E-03	3.79E-02
Activated CD8	CSF.1	0.33	4.52E-03	3.90E-02
Memory CD4	SCF	-0.33	4.65E-03	3.98E-02
CD4	SCF	0.32	4.85E-03	4.12E-02
Memory CD4	MCP.3	-0.32	5.08E-03	4.27E-02
Intermediate monocytes	IL6	0.32	5.16E-03	4.33E-02
CD3	EN.RAGE	-0.32	5.30E-03	4.42E-02
Monocytes	IL10	0.32	5.32E-03	4.43E-02
Activated CD8	CDCP1	0.32	5.50E-03	4.53E-02
CD4	MCP.3	0.32	5.58E-03	4.58E-02
Memory Treg	IL.17C	-0.32	5.63E-03	4.61E-02
Age	TNF	0.32	5.67E-03	4.63E-02

Variable 1	Variable 2	Spearman’s Rho	p-value	Adjusted p-value
Activated CD8	IL10	0.32	5.70E-03	4.64E-02
Activated CD8	IL.18R1	0.32	5.91E-03	4.76E-02
Classical monocytes	HGF	-0.32	5.98E-03	4.80E-02
Memory CD4	FGF.21	-0.32	6.07E-03	4.84E-02
Age	PD.L1	0.32	6.15E-03	4.89E-02

ESM Table 7 | List of genes and primers used for single cell gene expression profiling

Gene	Preamplification		qPCR	
	5'	3'	5'	3'
AHR	TAAAGCCAATCCCAGCTGAA	GACGCTGAGCCTAAGAACTGA	TAAAGCCAATCCCAGCTGAA	GACGCTGAGCCTAAGAACTGA
AURKA	GTCACAAGCCGGTTCAGAAT	TTTGATGCCAGTTCCTCCTC	GTCACAAGCCGGTTCAGAAT	TTTGATGCCAGTTCCTCCTC
BCL2	GAGTTCGGTGGGGTCATGT	ACAGCCAGGAGAAATCAAACA	GAGTTCGGTGGGGTCATGT	ACAGCCAGGAGAAATCAAACA
BCL6	AGCCGTGAGCAGTTTAGAGC	AAGTCCAGGAGGATGCAGAA	AGCCGTGAGCAGTTTAGAGC	AAGTCCAGGAGGATGCAGAA
CCL4	CTGTCCTGTCTCTCCTCATGC	GCTTGCTTCTTTTGGTTTGG	TAGCTGCCTTCTGCTCTCCA	GCTTGCTTCTTTTGGTTTGG
CCL5	CGCTGTCATCCTCATTGCTA	ACACACTTGGCGGTTCTTTC	ATCTGCCTCCCCATATTCCT	ACACACTTGGCGGTTCTTTC
CCR4	CAAATACAAGCGGCTCAGGT	AGCCCACCAAGTACATCCAG	CAAATACAAGCGGCTCAGGT	AGCCCACCAAGTACATCCAG
CCR5	GGCCATCTCTGACCTGTTTTT	AAACACAGCATGGACGACAG	GTCCCCTTCTGGGCTCACTA	AAACACAGCATGGACGACAG
CCR6	TCAGCGATGTTTTCGACTCC	CACCAGAATATTCCCCAGGA	TCAGCGATGTTTTCGACTCC	CACCAGAATATTCCCCAGGA
CCR7	CAATGAAAAGCGTGCTGGT	ATAGGGAGGAACCAGGCTTT	GTGGTGGCTCTCCTTGTCAT	ATAGGGAGGAACCAGGCTTT
CD247	GCACAGTTGCCGATTACAGA	TCAGGAACAAGGCAGTGAGA	GCACAGTTGCCGATTACAGA	TCAGGAACAAGGCAGTGAGA
CD27	CACTACTGGGCTCAGGGAAA	GCGAACGAGAAGACCAGAGT	CTCGTGAAGGACTGTGACCA	GCGAACGAGAAGACCAGAGT
CD3e	GCACTCACTGGAGAGTTCTGG	CCTCATCACCGCCTATGTTT	GCACTCACTGGAGAGTTCTGG	CCTCATCACCGCCTATGTTT
CD4	ACCGGGGAGTCCCTTTTAG	CATTCAGCTTGGATGGACCT	ACCGGGGAGTCCCTTTTAG	CATTCAGCTTGGATGGACCT
CD40LG	ATTGGGTCAGCACTTTTTGC	TTCACAAAGCCTTCAAACTGG	ATTGGGTCAGCACTTTTTGC	TTCACAAAGCCTTCAAACTGG
CD52	GCGCTTCCTCTTCCTCCTAC	CTGAAGCAGAAGAGGTGGATT	GCGCTTCCTCTTCCTCCTAC	CTGAAGCAGAAGAGGTGGATT
CD69	ATCCGGAGAGTGGACAAGAA	TGGTGATGAAGACCACATTCA	ATCCGGAGAGTGGACAAGAA	TGGTGATGAAGACCACATTCA
CD8B	GCTGGACTTCGCCTGTGATAT	TTGTCTCCCGATTTGACCAC	GCTGGACTTCGCCTGTGATAT	TTGTCTCCCGATTTGACCAC
CSF2	CACTGCTGCTGAGATGAATGA	AGGGCAGTGCTGCTTGTAGT	CACTGCTGCTGAGATGAATGA	AGGGCAGTGCTGCTTGTAGT
CTLA4	TGACAGCCAGGTGACTGAAG	GTTGCCTATGCCCAGGTAGT	TGGGGAATGAGTTGACCTTC	GTTGCCTATGCCCAGGTAGT
EGR1	CACCTGACCGCAGAGTCTTT	AGCGGCCAGTATAGGTGATG	CACCTGACCGCAGAGTCTTT	AGCGGCCAGTATAGGTGATG
Egr2	TGGAGAGAAGAGGTCGTTGG	GTTGAAGCTGGGGAAGTGAC	TGGAGAGAAGAGGTCGTTGG	GTTGAAGCTGGGGAAGTGAC
EOMES	CACAAATACCAACCCCGACT	GGGACAATCTGATGGGATGA	CACAAATACCAACCCCGACT	GGGACAATCTGATGGGATGA
FAS	CAAGGGATTGGAATTGAGGA	TGGAAGAAAAATGGGCTTTG	ATGGCCAATTCTGCCATAAG	TGGAAGAAAAATGGGCTTTG
FASLG	GGGATGTTTCAGCTCTTCCA	CAGAGGCATGGACCTTGAGT	CAGAAGGAGCTGGCAGAACT	CAGAGGCATGGACCTTGAGT
FASLG	GGCCTGTGTCTCCTTGTGAT	GTGGCCTATTTGCTTCTCCA	GGGATGTTTCAGCTCTTCCA	GTGGCCTATTTGCTTCTCCA
FOSl	CCGGGGATAGCCTCTCTTAC	ACTGGTCGAGATGGCAGTG	ACTACCACTCACCCGCAGAC	ACTGGTCGAGATGGCAGTG
FOSL2	CAGCAGAAATTCCGGGTAGA	GGTATGGGTTGGACATGGAG	CAGCAGAAATTCCGGGTAGA	GGTATGGGTTGGACATGGAG

ESM Table 7 continued

	Preamplification		qPCR	
Gene	5'	3'	5'	3'
FOXO1	AAGAGCGTGCCCTACTTCAA	TTCCTTCATTCTGCACACGA	AAGAGCGTGCCCTACTTCAA	TTCCTTCATTCTGCACACGA
FOXP3	GTAGCCATGGAAACAGCACAT	GCGTGTGAACCAGTGGTAGAT	ACATTCCCAGAGTTCCTCCAC	GCGTGTGAACCAGTGGTAGAT
GZMA	GAACAAAAGGTCCCAGGTCA	TTTTTGCTTTTTCCATCAGC	GAACAAAAGGTCCCAGGTCA	TTTTTGCTTTTTCCATCAGC
GZMB	GGTGGCTTCCTGATACGAGA	GCTGCAGTAGCATGATGTCG	ACTGTTGGGGAAGCTCCATA	GCTGCAGTAGCATGATGTCG
GZMH	CAGCCATTCCTCCTCCTGT	GAGCAGCTGTCAGCACAAAG	TCCTCCTGTTGGCCTTTCTT	GAGCAGCTGTCAGCACAAAG
H2AFX	TACCTCACCGCTGAGATCCT	AGCTTGTTGAGCTCCTCGTC	TACCTCACCGCTGAGATCCT	GTTGAGCTCCTCGTCGTTG
HMGB1	AAGCACCCAGATGCTTCAGT	TCCGCTTTTGCCATATCTTC	AAGCACCCAGATGCTTCAGT	TCCGCTTTTGCCATATCTTC
ICOS	GGACCATTCTCATGCCAACT	TCGTGCACACTGGATGAATA	GGTTACCCATAGGATGTGCAG	TCGTGCACACTGGATGAATA
IFNA1	ACCCACAGCCTGGATAACAG	ACTGGTTGCCATCAAACTCC	ACCCACAGCCTGGATAACAG	ACTGGTTGCCATCAAACTCC
IFNAR1	TGAGTCTGTCGGGAATGTGA	TGCGAAATGGTGTAAATGAGTC	TGAGTCTGTCGGGAATGTGA	TGCGAAATGGTGTAAATGAGTC
IFR8	ACGAGGTTACGCTGTGCTTT	TGATCAGCTCGTCGATTTCA	ACGAGGTTACGCTGTGCTTT	TGATCAGCTCGTCGATTTCA
IKZF2	CGAAAGGGAGCACTCCAATA	ATGGCCCCTGATCTCATCT	CGAAAGGGAGCACTCCAATA	ATGGCCCCTGATCTCATCT
IL10	TGCTGGAGGACTTTAAGGGTTA	GCCTTGCTCTTGTTTTCACAG	TTTAAGGGTTACCTGGGTTGC	GCCTTGCTCTTGTTTTCACAG
IL13	GGTCAACATCACCCAGAACC	TTTACAAACTGGGCCACCTC	GTACTGTGCAGCCCTGGAAT	TTTACAAACTGGGCCACCTC
IL17A	TGGGAAGACCTCATTGGTGT	CCGGTTATGGATGTTCAGGT	TGGGAAGACCTCATTGGTGT	CCGGTTATGGATGTTCAGGT
IL17f	TCCAAAAGCCTGAGAGTTGC	ATGCAGCCCAAGTTCCTACA	GCCTGTGCCAGGAGGTAGTA	ATGCAGCCCAAGTTCCTACA
IL18RAP	TTGCAGGAGAGCGAATTAAA	GGTGAGAGTCGATTTCTGTGG	TTGCAGGAGAGCGAATTAAA	GGTGAGAGTCGATTTCTGTGG
IL2	TGGAGCATTTACTGCTGGATT	GCACTTCCTCCAGAGGTTTG	TGGAGCATTTACTGCTGGATT	GCACTTCCTCCAGAGGTTTG
IL21	TCGCCACATGATTAGAATGC	AAGCAGGAAAAAGCTGACCA	TCGCCACATGATTAGAATGC	AAGCAGGAAAAAGCTGACCA
IL22	TCCAGCAGCCCTATATCACC	GTTCAGCACCTGCTTCATCA	TCCAGCAGCCCTATATCACC	GTTCAGCACCTGCTTCATCA
IL2RA	ACTGCTCACGTTCATCATGG	CGGAAACCTCTCTTGCATTC	ACTGCTCACGTTCATCATGG	CGGAAACCTCTCTTGCATTC
IL4	TGCCTCCAAGAACACAACTG	CTCTGGTTGGCTTCCTTCAC	GGCAGTTCTACAGCCACCAT	CTCTGGTTGGCTTCCTTCAC
IL6R	CTCAGTGTCACCTGGCAAGA	CCTTGACCATCCATGTTGTG	CTCAGTGTCACCTGGCAAGA	CCTTGACCATCCATGTTGTG
IL7R	CTGAGGCTCCTTTTGACCTG	CTGCAGGAGTGTCAGCTTTG	CTGAGGCTCCTTTTGACCTG	CTGCAGGAGTGTCAGCTTTG
IL9	CTCATCAACAAGATGCAGGAAG	TGTTTGCATGGTGGTATTGG	CTCATCAACAAGATGCAGGAAG	TGTTTGCATGGTGGTATTGG
INFg	CTGTTACTGCCAGGACCCAT	TGGATGCTCTGGTCATCTTT	GGTCATTCAGATGTAGCGGA	TGGATGCTCTGGTCATCTTT
IRF3	GTTCTGTGTGGGGGAGTCAT	CCTTGTACTGGTCGGAGGTG	GTTCTGTGTGGGGGAGTCAT	CCTTGTACTGGTCGGAGGTG
JAK3	GAATGGTGCCCAGCTCTATG	TGAAAGTCCCTCTGCTGGTC	GAATGGTGCCCAGCTCTATG	TGAAAGTCCCTCTGCTGGTC

ESM Table 7 continued

	Preamplification		qPCR	
Gene	5'	3'	5'	3'
JUN	CCCCAAGATCCTGAAACAGA	CCGTTGCTGGACTGGATTAT	CCCCAAGATCCTGAAACAGA	CCGTTGCTGGACTGGATTAT
MAF	GGACGCGTACAAGGAGAAAT	GCTTCCAAAATGTGGCGTAT	GGACGCGTACAAGGAGAAAT	GCTTCCAAAATGTGGCGTAT
NAB2	CATCTATGGCCGTTTCGACT	GTGCTCTCTCGGGCTACTTG	CATCTATGGCCGTTTCGACT	GTGCTCTCTCGGGCTACTTG
PRF	AACTTTGCAGCCCAGAAGAC	GGGTGCCGTAGTTGGAGATA	ACAGCTTCAGCACTGACACG	GGGTGCCGTAGTTGGAGATA
REL	ACAAATGTGAAGGGCGATCA	CCGTCTCTGCAGTCTTTTCC	GGAGCACAGCACAGACAACA	CCGTCTCTGCAGTCTTTTCC
RGS16	CACGCTTTCCTGAAGACAGA	GACCTCTTTAGGGGCCTCAC	CACGCTTTCCTGAAGACAGA	GACCTCTTTAGGGGCCTCAC
RORA	CACCAGCATCAGGCTTCTTT	GGTCTGCCACGTTATCTGCT	CACCAGCATCAGGCTTCTTT	GGTCTGCCACGTTATCTGCT
RUNX1	CCCTAGGGGATGTTCCAGAT	TGAAGCTTTTCCCTCTTCCA	CCCTAGGGGATGTTCCAGAT	TGAAGCTTTTCCCTCTTCCA
RUNX2	CGGAATGCCTCTGCTGTTAT	TGGGGAGGATTTGTGAAGAC	CGGAATGCCTCTGCTGTTAT	TGGGGAGGATTTGTGAAGAC
SRP14	TATGACGGTCGAACCAAACC	GCTGCTGCTTTGGTCTTCTT	TACTGTGGAGGGCTTTGAGC	GCTGCTGCTTTGGTCTTCTT
STAT3	GCTTTTGTCAGCGATGGAGT	GCTGCAACTCCTCCAGTTTC	GCTTTTGTCAGCGATGGAGT	GCTGCAACTCCTCCAGTTTC
TBX21	CCGTGACTGCCTACCAGAAT	ATCTCCCCCAAGGAATTGAC	CCGTGACTGCCTACCAGAAT	ATCTCCCCCAAGGAATTGAC
TGFB1	TACCTGAACCCGTGTTGCT	CACAACTCCGGTGACATCAAA	TACCTGAACCCGTGTTGCTT	CAACTCCGGTGACATCAAAA
TGFBR2	TCCACCTGTGACAACCAGAA	GGAGAAGCAGCATCTTCCAG	ATGAGCAACTGCAGCATCAC	GGAGAAGCAGCATCTTCCAG
TMEM2	TTGCCCAGATCAAAATCCTC	TCCCCAAATACAAGCAGTCC	TCTCAGGAATTGGGATCCAG	TCCCCAAATACAAGCAGTCC
TNF	CCCCAGGGACCTCTCTCTAA	TGAGGTACAGGCCCTCTGAT	CCCGAGTGACAAGCCTGTAG	TGAGGTACAGGCCCTCTGAT
TNFRSF18	CACTGCAAACCTTGGACAGA	CCACATGCACTGACTCCTCA	CACTGCAAACCTTGGACAGA	CCACATGCACTGACTCCTCA
TNFRSF9	CACTCTGTTGCTGGTCCTCA	CCTGGTCCTGAAAACACCTT	CACTCTGTTGCTGGTCCTCA	CCTGGTCCTGAAAACACCTT
TNFSF10	GACAGACCTGCGTGCTGAT	CAGCAGGGGCTGTTCATACT	CCTGCAGTCTCTCTGTGTGG	CAGCAGGGGCTGTTCATACT
UBE2C	TGGCGATAAAGGGATTTCTG	GGCGTGAGGAACTTCACTGT	TTTCAAATGGGTAGGGACCA	GGCGTGAGGAACTTCACTGT

ESM Fig. 1

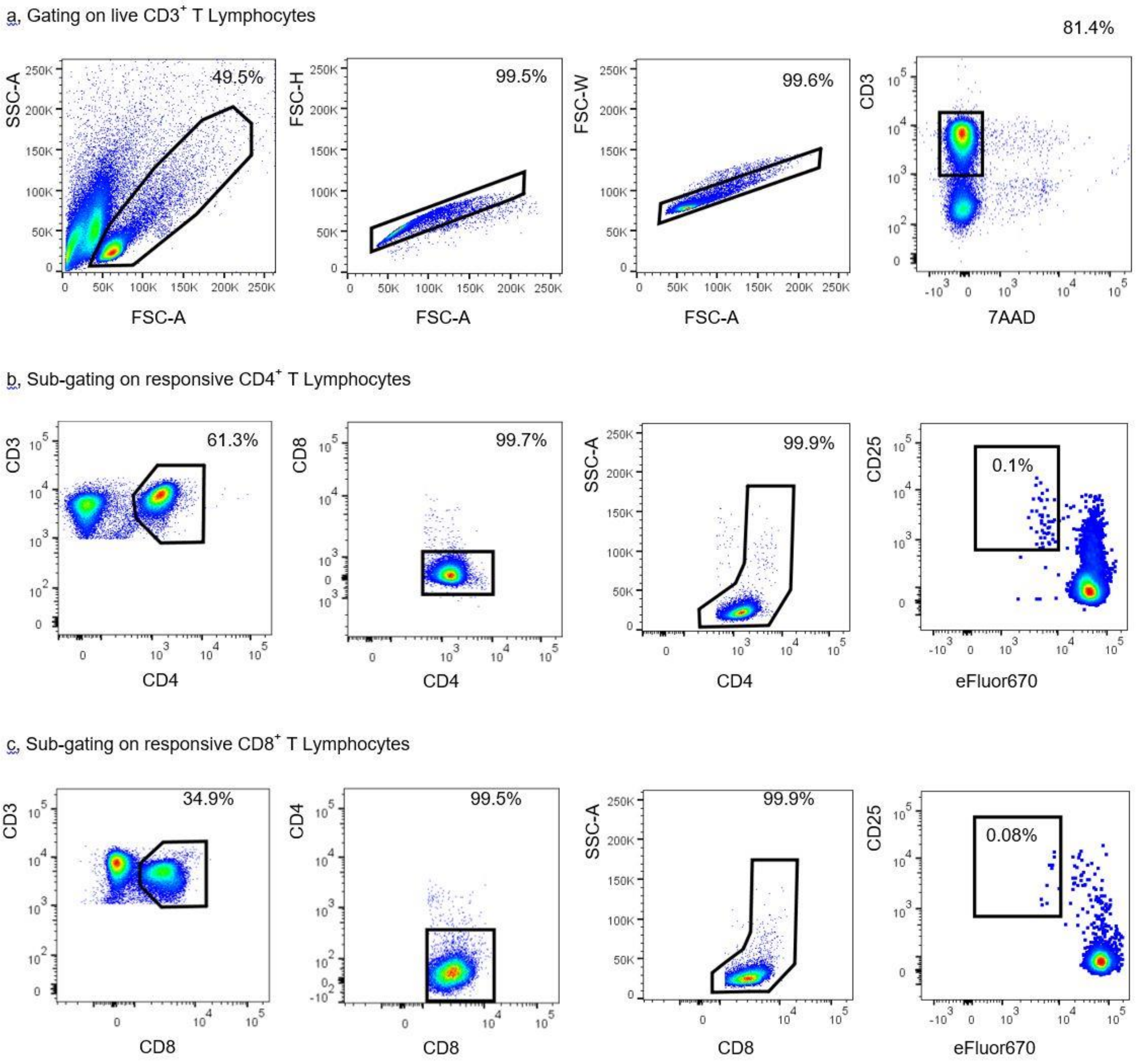

ESM Fig. 1 | Flow analysis scheme to identify insulin responsive T cells. Flow analysis was performed after 5-day culture on eFluor670 dye-labelled PBMC incubated in the presence of 50 µg/ml insulin. a, Gating strategy used to identify live single $CD4^+$ T lymphocytes. b, Sub-gating on $CD4^+$ T lymphocytes and identification of eFluor670dimCD25high responsive $CD4^+$ T cells. c, Sub-gating on $CD8^+$ T lymphocytes and identification of eFluor670dimCD25high responsive $CD8^+$ T cells.

ESM Fig. 2

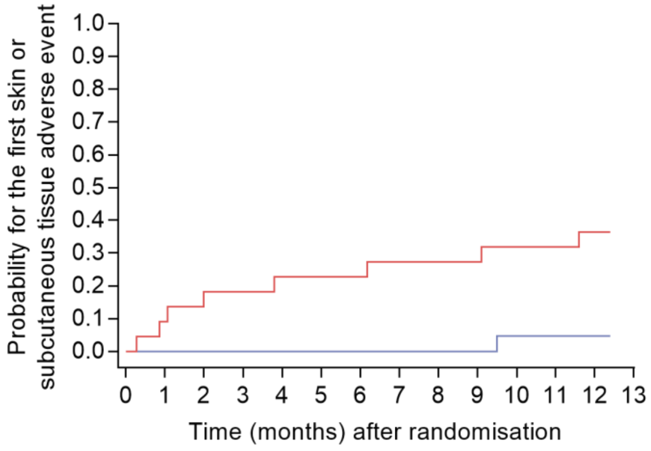

ESM Fig. 2 | Skin or subcutaneous tissue disorders. Significant difference (p = 0.011) in the cumulative probability for the first skin or subcutaneous tissue adverse event in children treated with placebo (blue line) or oral insulin (red line).

ESM Fig. 3

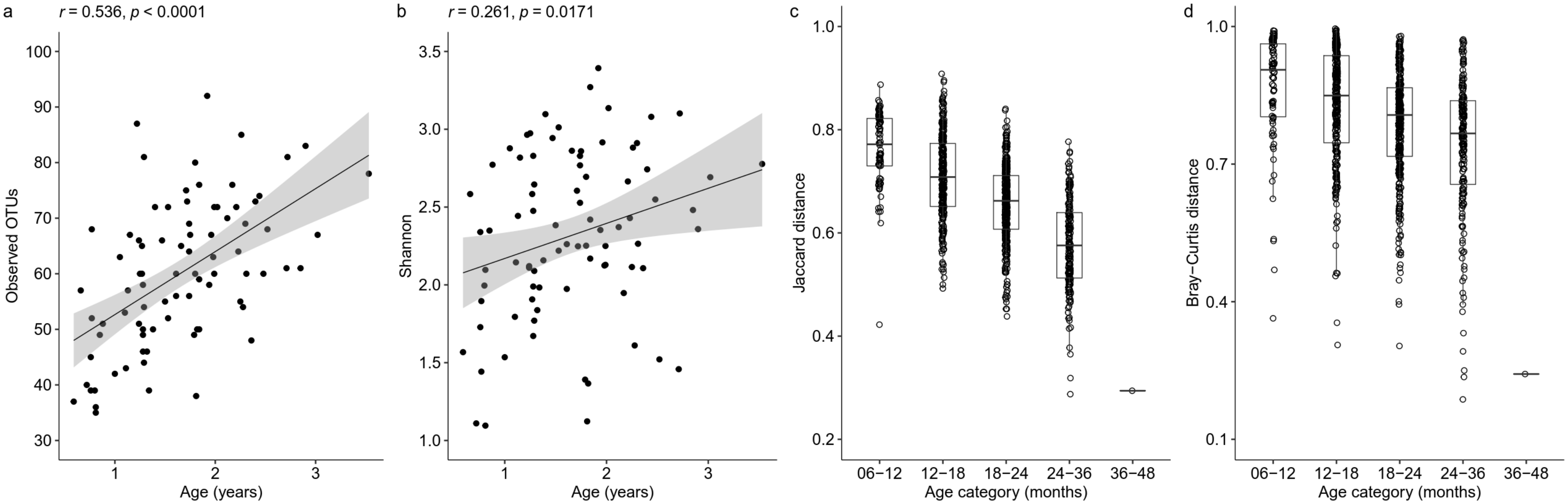

ESM Fig. 3 | Microbiome alterations in relation to age by WGS. a,b, Alpha diversity in relation to age over the time of study participation. a, Richness (Observed OTUs) and, b, evenness (Shannon). c,d, Beta diversity in relation to age over time of study participation. c, Jaccard distance, d, Bray-Curtis distance where each dot represents the distance between two samples within the age range.

ESM Fig. 4

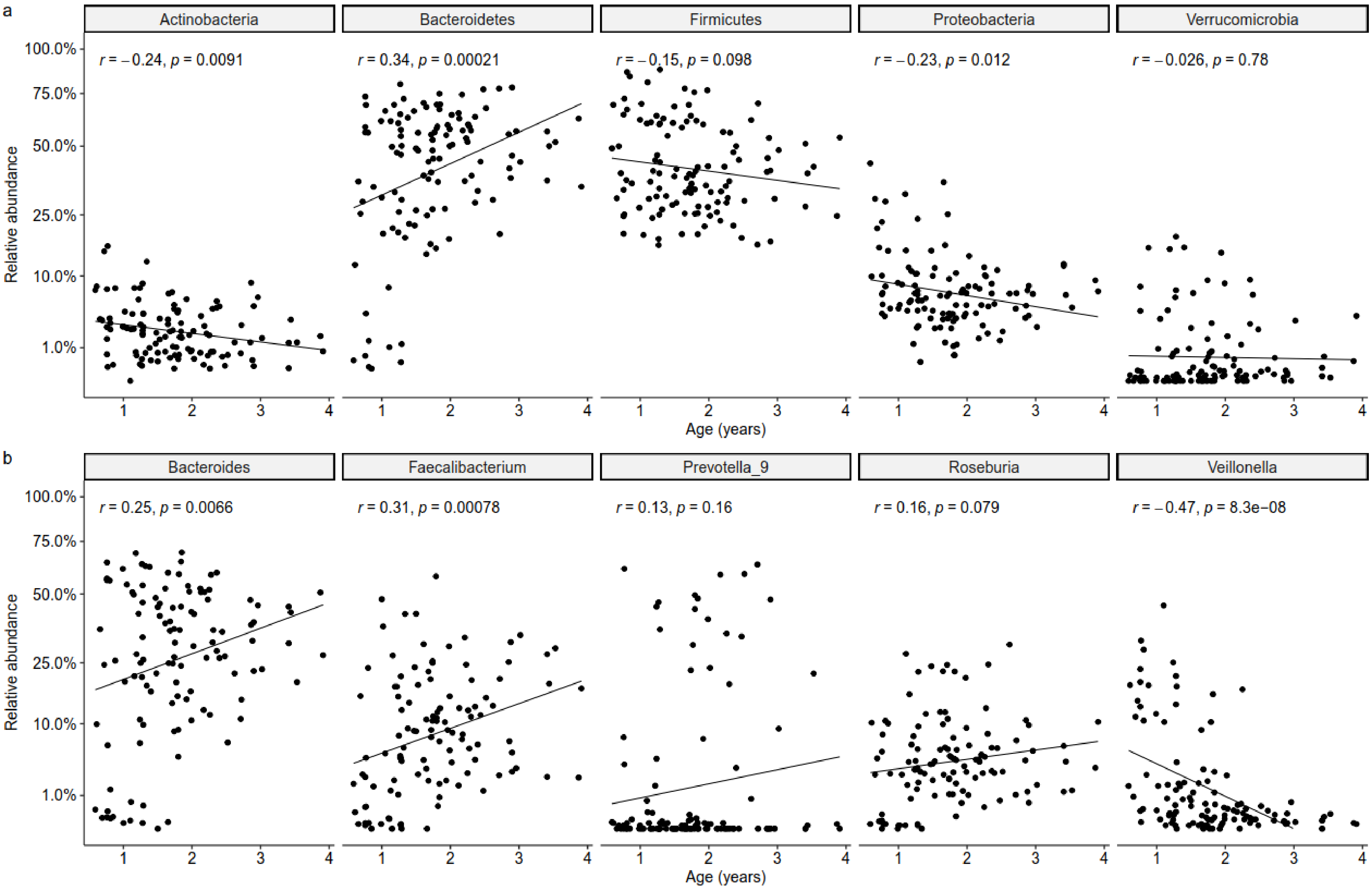

ESM Fig. 4 | Relative abundance of species level taxonomies in relation to age. a,b, the five most expressed phyla (a), and genera (b).

ESM Fig. 5

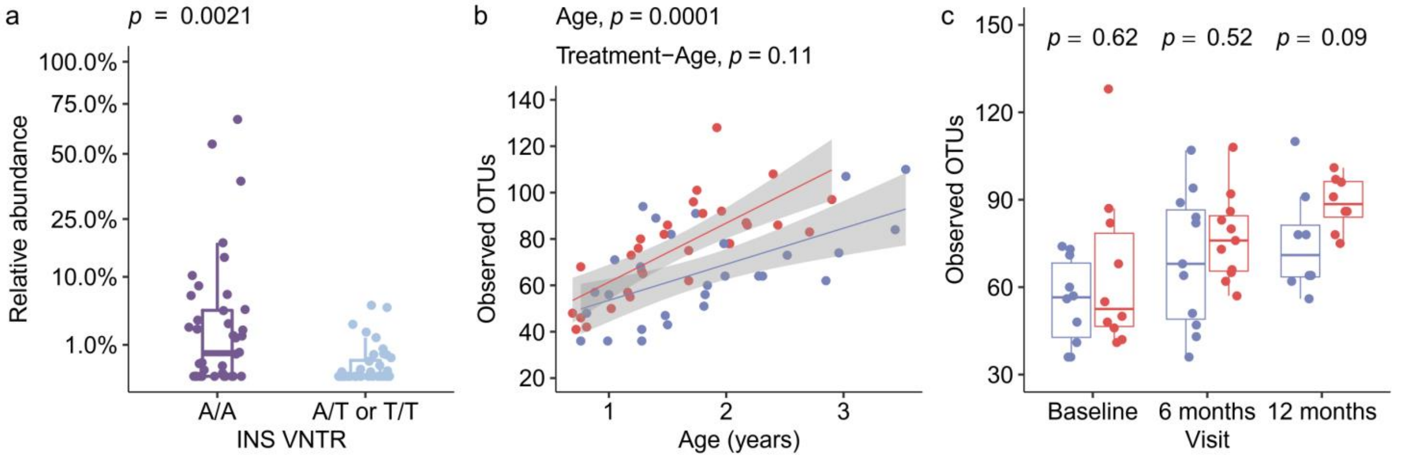

ESM Fig. 5 | Microbiome alterations in relation to age, treatment, and genotype. a, Relative abundance of Bacteroides dorei (baseline, 6 and 12 months) in children with the *INS AA* genotype (purple circles, *n* = 40 samples from 16 children) or *INS AT* or *TT* genotypes (green circles, *n* = 40 samples from 15 children). b, Alpha diversity by observed OTUs in relation to age over the time of study participation (baseline, 6 and 12 months) in children who received placebo (blue line and dots, *n* = 29 samples from 11 children) or oral insulin (red line and dots, *n* = 29 samples from 11 children). c, Alpha diversity by observed OTUs at baseline, 6 and 12 months in children who received placebo (blue dots, *n* = 10 at baseline, 11 at 6 months, 8 at 12 months) or oral insulin (red dots, *n* = 10 at baseline, 11 at 6 months, 8 at 12 months).

ESM Fig. 6

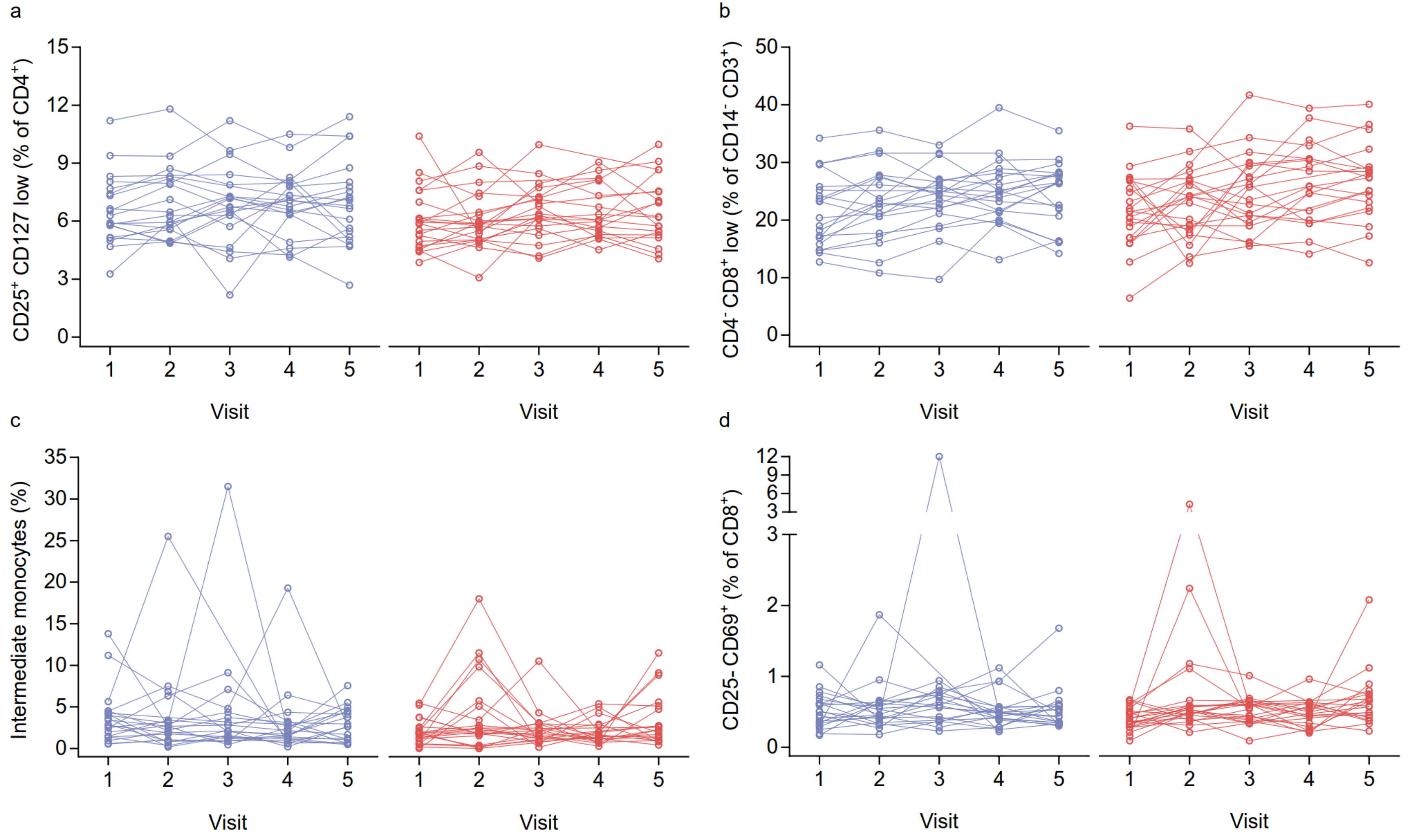

ESM Fig. 6 | Immune cell population frequencies over time of study participation in children receiving placebo (blue lines; *N* = 21) or oral insulin (red lines; *N* = 22).

a, $CD4^{+}$ regulatory T cells. b, $CD8^{+}$ T cells. c, intermediate monocytes ($CD14^{++}CD16^{+}$ monocytes). d, $CD69^{+}$ activated $CD8^{+}$ T cells.

ESM Fig. 7

a Correlation between cell population frequencies

b Age prediction from cell frequencies

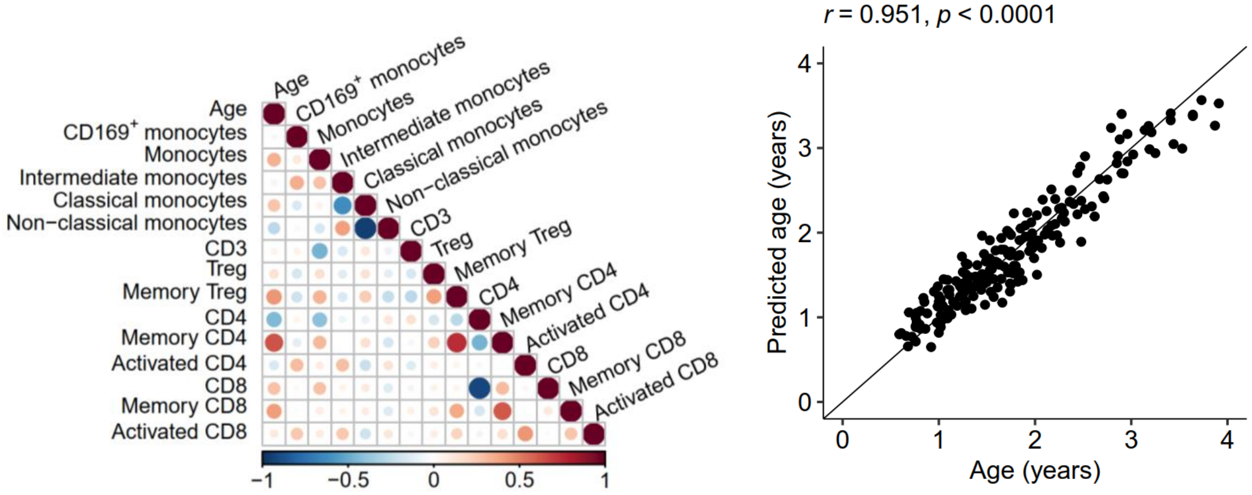

ESM Fig. 7 | Immune profiling markers in the study participants. a, Correlations between cell population frequencies and age for all study visits (N = 208 samples from 43 children). b, Age predicted by a function of the cell population frequencies shown in a as compared to the actual age of children when samples were drawn.

ESM Fig. 8

a OTU diversity b Shannon diversity c Age

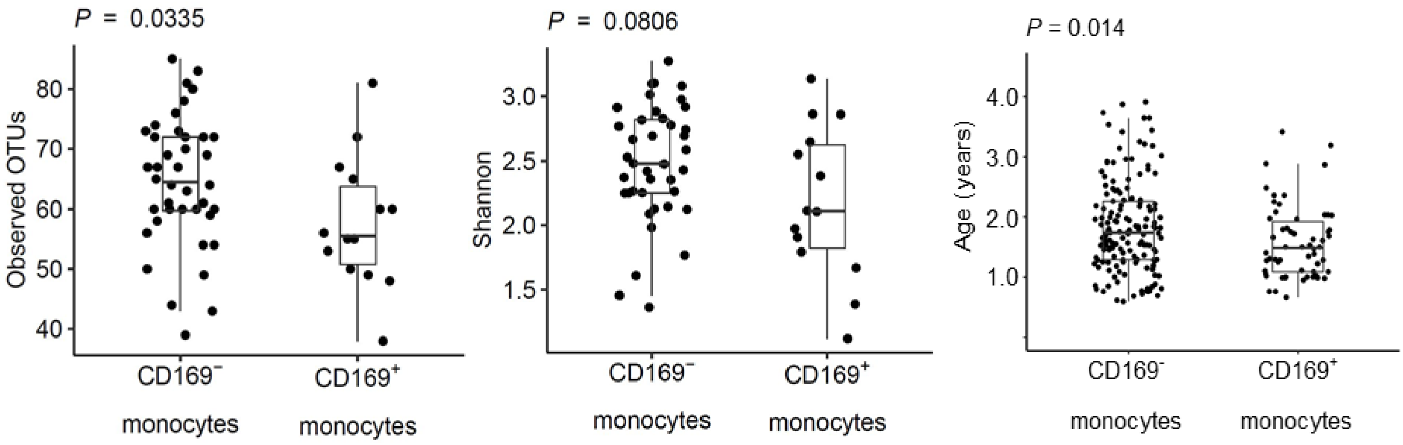

ESM Fig. 8 | Monocyte CD169 expression associations with microbiome and age. a, Alpha diversity richness in stool microbiome (OTUs) in samples at baseline, 6 and 12 months that were monocyte CD169 negative ($CD169^-$) or positive ($CD169^+$). b, Alpha diversity evenness in stool microbiome (Shannon index) in samples at baseline, 6 and 12 months that were monocyte CD169 negative ($CD169^-$) or positive ($CD169^+$). c, Age of participant at sample draw for 148 samples that were monocyte CD169 negative ($CD169^-$) and 57 samples that were monocyte CD169 positive ($CD169^+$).

ESM Fig. 9

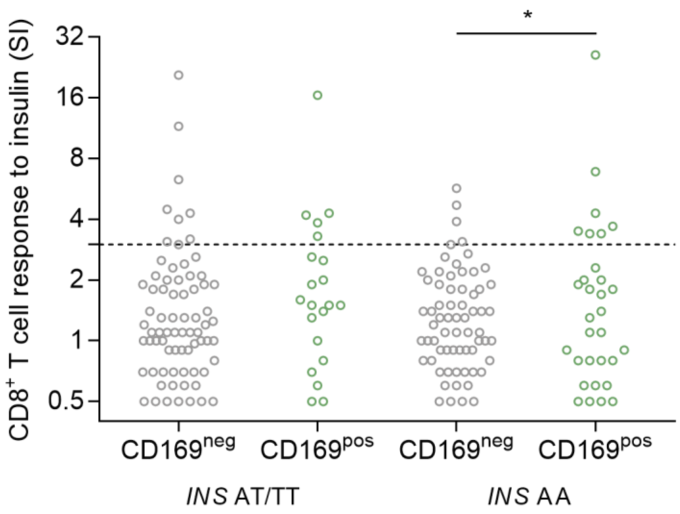

ESM Fig. 9 | CD8+ T cell responses to insulin (SI) in samples from 20 children with the *INS AT* or *TT* genotype and 22 children with the *INS AA* genotype and stratified by monocyte CD169 expression as negative (CD169neg, black circles; $n = 148$ samples) or positive (CD169pos, green circles; $n = 58$ samples) in all study visits. * $p < 0.05$

ESM Fig. 10

a

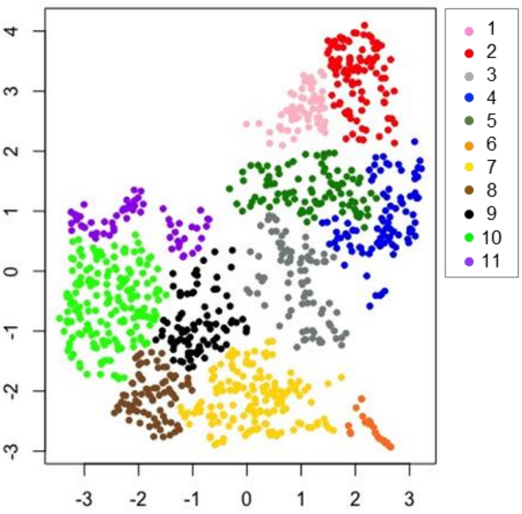

b

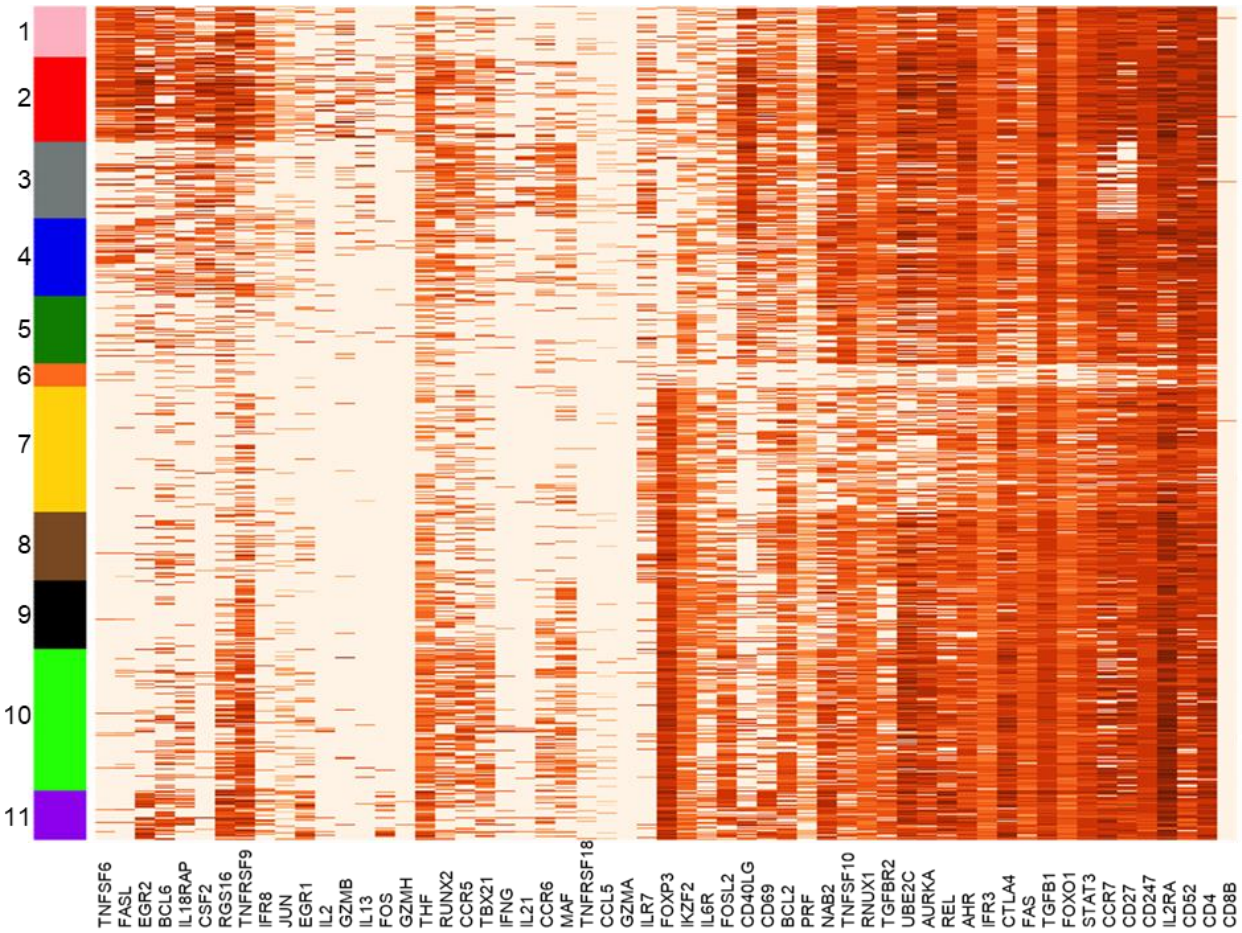

ESM Fig. 10 | Gene expression in insulin-responsive CD4$^+$ T cells. a, UMAP of expression for 76 genes in 1036 insulin-responsive CD4$^+$ T cells. Each dot represents a cell. The cells are distributed to 11 clusters containing 61 (cluster 1), 102 (cluster 2), 82 (cluster 3), 93 (cluster 4), 93 (cluster 5), 28 (cluster 6), 151 (cluster 7), 82 (cluster 8), 82 (cluster 9), 171 (cluster 10), and 60 (cluster 11) cells. b, Heatmap of gene expression for the genes that differed in their expression between the 11 clusters identified in supplementary Fig. 8a. Clusters are arranged from 1 to 11 vertically. Cluster 2 (red) and 3 (gray) were considered Th1/Th21-like. Cluster 2 cells often expressed CSF2, TNF, TBX21 and IFNG, which are characteristic of Th1 cells, and many cluster 3 cells also expressed IL21. Clusters 9 (black), 10 (light green), and 11 (purple) were considered Treg-like due to their strong FOXP3 and IKZF2 expression, and lack or sparseness of CD127 and cytokine gene expression.
